# CRISPR-based oligo recombineering prioritizes apicomplexan cysteines for drug discovery

**DOI:** 10.1038/s41564-022-01249-y

**Published:** 2022-10-20

**Authors:** H. J. Benns, M. Storch, J. A. Falco, F. R. Fisher, F. Tamaki, E. Alves, C. J. Wincott, R. Milne, N. Wiedemar, G. Craven, B. Baragaña, S. Wyllie, J. Baum, G. S. Baldwin, E. Weerapana, E. W. Tate, M. A. Child

**Affiliations:** 1 Department of Life Sciences, Imperial College London, London, UK; 2 Department of Chemistry, Imperial College London, London, UK; 3 London Biofoundry, Imperial College Translation & Innovation Hub, London, UK; 4 Department of Chemistry, Boston College, Boston, MA USA; 5 Wellcome Centre for Anti-Infectives Research, School of Life Sciences, University of Dundee, Dundee, UK; 6 School of Biomedical Sciences, UNSW, Sydney, NSW Australia

**Keywords:** Target identification, Parasitology, Mutagenesis, Proteomics

## Abstract

Nucleophilic amino acids are important in covalent drug development yet underutilized as anti-microbial targets. Chemoproteomic technologies have been developed to mine chemically accessible residues via their intrinsic reactivity towards electrophilic probes but cannot discern which chemically reactive sites contribute to protein function and should therefore be prioritized for drug discovery. To address this, we have developed a CRISPR-based oligo recombineering (CORe) platform to support the rapid identification, functional prioritization and rational targeting of chemically reactive sites in haploid systems. Our approach couples protein sequence and function with biological fitness of live cells. Here we profile the electrophile sensitivity of proteinogenic cysteines in the eukaryotic pathogen *Toxoplasma gondii* and prioritize functional sites using CORe. Electrophile-sensitive cysteines decorating the ribosome were found to be critical for parasite growth, with target-based screening identifying a parasite-selective anti-malarial lead molecule and validating the apicomplexan translation machinery as a target for ongoing covalent ligand development.

## Abstract (web summary)

A CRISPR-based oligo recombineering (CORe) approach functionally prioritizes electrophile-sensitive cysteines in *Toxoplasma gondii* and promotes anti-parasitic drug discovery.

## Main

Electrophilic small molecules that engage protein-encoded amino-acid nucleophiles are resurgent in drug discovery as versatile chemical probes and therapeutic agents^1,2^. As a result, considerable efforts are devoted to the development of chemical proteomic technologies termed ‘reactivity-based profiling’ for the identification of nucleophilic sites^3^. Isotopic tandem-orthogonal activity-based protein profiling (isoTOP-ABPP) is a standard method for proteome-wide profiling of intrinsic amino-acid reactivity^4^. Central to isoTOP-ABPP is the use of quantitative mass spectrometry (MS) to measure the extent of protein labelling with a highly reactive electrophilic probe. Initially applied to rank the reactivity of cysteines in the human proteome using an iodoacetamide (IAA) probe^4^, isoTOP-ABPP has since been expanded to other amino-acid types, including lysine^5^, methionine^6^ and tyrosine^7^. Moreover, this method has been successfully adapted for competitive screening of covalent fragments^5,8–10^, enabling identification of sites that can be pursued in fragment-based lead discovery programmes for ‘inverse drug discovery’^11^.

Despite advances in reactivity-based profiling, the prioritization of chemically reactive or ligandable amino acids as targets following their proteomic identification remains biased; target selection is typically based on the availability of existing functional information or assays for the associated protein class. This inevitably leads to proteins with untapped therapeutic value being overlooked^12^. The ability to efficiently interrogate individual amino acids across the proteome at high throughput would expand our understanding of protein sequence–function relationships in complex biological systems, and solve one of the grand challenges of universal inverse drug discovery.

Over recent years, multiplexed ‘recombineering’ screens (for example, MAGE, CRMAGE and CREATE) have been developed to simultaneously map the phenotypic effects of thousands of amino-acid substitutions across genomes^13–15^. These platforms monitor the allelic frequency of amino-acid mutants in a population over a period of selective pressure or growth, enabling the identification of substitutions that impact cellular fitness. Such methods typically indirectly estimate mutant frequency, limiting their ability to probe sequence–function relationships. Other technologies overcome these limitations by directly sequencing the modified chromosomal loci^16–18^. However, their application has been restricted to single or small panels of targets (for example, isolated exons or individual loci in saturation mutagenesis) and/or a limited range of amino-acid substitution types. Therefore, a strategy for direct, quantitative assessment of the contribution of individual amino acids to protein function across diverse genomic loci (such as sites identified by reactivity-based profiling or post-translationally modified protein networks) is needed.

In this Article, we introduce CRISPR-based oligo recombineering (CORe) for proteome-wide assessment of amino-acid contribution to protein function in cells. Combined with isoTOP-ABPP, we apply CORe to the eukaryotic pathogen *Toxoplasma gondii*, functionally annotating electrophile-sensitive cysteines and prioritizing new therapeutic targets. Validating our strategy, we leverage *T. gondii*’s evolutionary relationship with the malaria parasite, *Plasmodium falciparum*, and confirm that cysteine-targeted electrophilic fragments can inhibit *P. falciparum* protein translation. Our work introduces CORe for scalable protein sequence–function studies and the expedient, unbiased prioritization of chemically reactive sites, proteins and biological processes for ligand discovery.

## Cysteine reactivity profiling in *T. gondii*

We sought to establish a platform for the prioritization of covalently druggable sites on protein targets. While we intended our final CORe target prioritization platform to be amino acid agnostic, for proof of concept we focused on electrophile-sensitive cysteines in the apicomplexan parasite *T. gondii*. *T. gondii* is an experimentally tractable eukaryotic host–pathogen model^19^ with medical and veterinary importance^20^. However, current frontline therapeutics are ineffective against the chronic bradyzoite lifecycle stage and limited by toxicity^21^, highlighting the need for rapid identification and prioritization of new therapeutic targets.

**Fig. 1:**
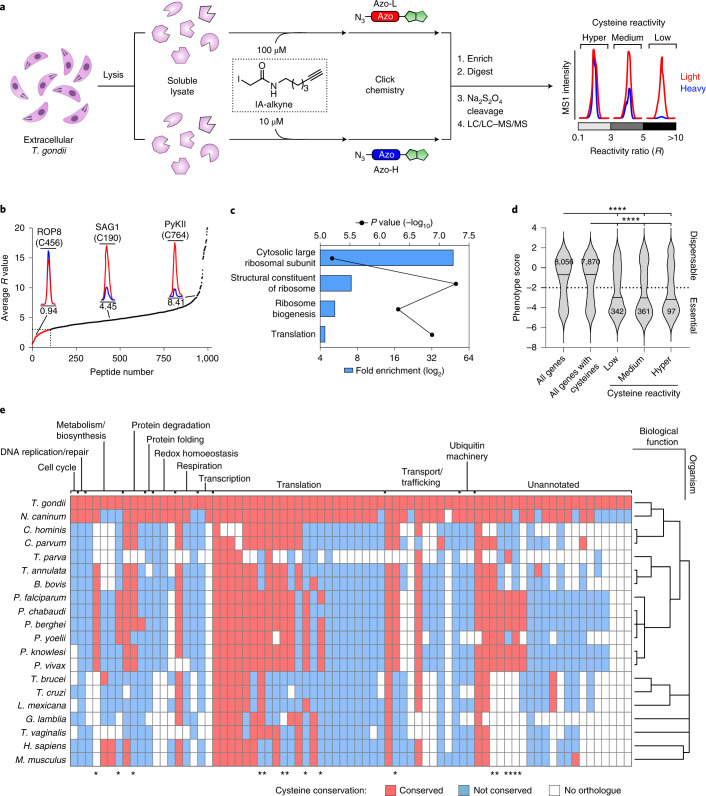
Cysteine reactivity profiling in *T. gondii* reveals enrichment of highly electrophile-sensitive cysteines in essential and translation-associated proteins. **a**, isoTOP-ABPP workflow for quantifying cysteine electrophile-sensitivity in *T. gondii* parasites. Soluble lysates from extracellular tachyzoites were independently labelled with high (100 µM) and low (10 µM) concentrations of a thiol-reactive IA-alkyne probe. Labelled samples were then click-conjugated to isotopically differentiated, reductant-cleavable biotin tags (heavy (blue) and light (red) for 10 µM and 100 µM treatment groups, respectively), combined and enriched on streptavidin-immobilized beads. Immobilized proteins were then subject to tandem on-bead trypsin digestion and sodium hydrosulfite treatment, eluting probe-modified peptides for LC/LC–MS/MS analysis. Cysteine electrophile sensitivity is quantified by *R* values, which represent the differences in MS1 peak intensities between the light- and heavy-conjugated proteomes. **b**, Ranked average *R* values for probe-labelled peptides from two independent experiments (*n* = 2). Representative chromatograms of cysteines within three groups of reactivity (high, *R* < 3; medium, *R* = 3–5; low, *R* > 5) are annotated. **c**, Enrichment analysis of functional annotations in annotated genes containing highly electrophile-sensitive cysteines relative to the *T. gondii* genome. Fold change is plotted against statistical significance determined from a two-tailed Fisher’s exact test. **d**, Comparative distribution analysis of published phenotype scores^25^ for the *T. gondii* tachyzoite genome with all cysteine- and electrophile-sensitive cysteine-containing genes. Essential genes are classified by a score of <−2. Statistical significance was assessed using two-tailed Kolmogorov–Smirnov *t*-test (****P < 0.0001). **e**, Conservation of highly electrophile-sensitive cysteines identified in essential *T. gondii* genes across eukaryotic orthologues in *Neospora caninun*, *Cryptosporidium hominis*, *Cryptosporidium parvum*, *Theileria parva*, *Theileria annulata*, *Babesia bovis*, *Plasmodium chabaudi*, *Plasmodium berghei*, *Plasmodium yoelii*, *Plasmodium knowlesi*, *Plasmodium vivax*, *Trypanosoma brucei*, *Trypanosoma cruzi*, *Leishmania mexicana*, *Giardia lamblia*, *Trichomonas vaginalis*, *Homo sapiens*, *Mus musculus*.. Cysteines are grouped by the predicted function of their associated genes, and organisms by their phylogenetic relationship. Asterisks indicate residues highly conserved in eukaryotic pathogens, but absent in mammalian systems. Source data

**Extended Data Fig. 1:**
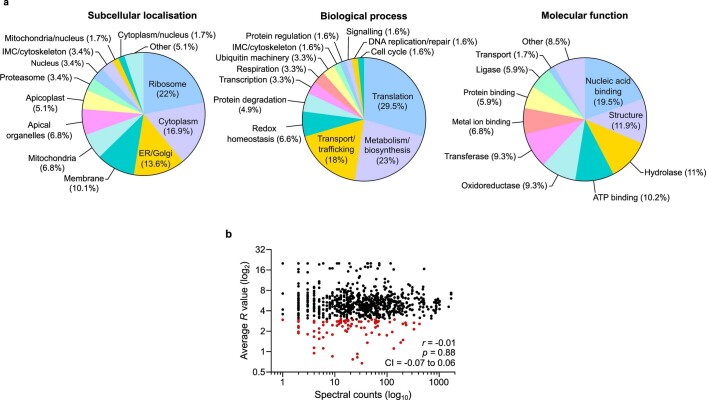
Enrichment hyperreactive cysteines in ribosomal proteins is independent of protein abundance. **a**, Proportions of hyperreactive cysteine-containing genes with functional annotations for three gene ontology categories; subcellular localization, biological process and molecular function. Pie charts depict overrepresentation of naturally abundant proteins, such as ribosome components. **b**, Correlation of isoTOP-ABPP *R* values against total spectral counts of the associated proteins (a semi-quantitative measure of protein abundance). Spectral counts were obtained from a published proteomic dataset for extracellular *T. gondii* parasites^64^. Annotated *r* values indicate the degree of correlation between the datasets following a two-tailed Pearson’s correlation analysis. Annotated *p* and CI values reflect the statistical significance and 95% confidence intervals of the correlation analysis, respectively. Note that proteins with low *R* values (< 3, highlighted in red) span a broad range of spectral counts. Source data

We applied a variant of the isoTOP-ABPP platform that uses an iodoacetamide-alkyne (IA-alkyne) probe and isotopically differentiated biotin tags with cleavable azobenzene linkers^22^ to *T. gondii* (Fig. 1a). We identified a total of 1,097 cysteines in 691 proteins that were labelled by IA-alkyne in extracellular *T. gondii* tachyzoites (Supplementary Tables 1 and 2). Similarly to previous studies^4,23,24^, individual cysteines displayed a range of inherent reactivity towards the probe (Fig. 1b). Amino-acid ‘hyperreactivity’ was previously found to be a predictor of functionality in cells^4,5^, and we hypothesized the same might be true in *T. gondii*. We therefore partitioned cysteines by their respective isotopic ratios into high (*R* < 3), medium (*R* = 3–5) and low (*R* > 5) reactivity groups. As reactivity is a relative concept determined by the electrophilic component of the probe, we refer to the spread of residue reactivity in terms of electrophile sensitivity from hereon. In total, 130 highly electrophile-sensitive cysteines were identified in 97 proteins with diverse biological functions (Extended Data Fig. 1a). This includes proteins with known cysteine-based catalytic mechanisms (for example, thioredoxins), well-characterized parasite proteins for which no functional role has previously been attributed to the identified cysteines (for example, myosin F) and hypothetical proteins (Supplementary Table 3). Analysis of functional annotations assigned to highly electrophile-sensitive cysteine-containing genes revealed enrichment of translation-associated proteins, including those of the ribosome (Fig. 1c, Extended Data Fig. 1a and Supplementary Table 4), which were not correlated with protein abundance (Extended Data Fig. 1b) and were absent from similar datasets obtained from other eukaryotic cell systems^4,23,24^.

We next assessed the association of highly electrophile-sensitive cysteines with gene essentiality according to ‘phenotype scores’ from a genome-wide CRISPR knockout (KO) screen in *T. gondii* (Fig. 1d)^25^. The more negative a gene’s phenotype score, the more fitness conferring the gene’s function is in vitro. Using a phenotype score threshold of −2 or below as an indicator of gene essentiality, we observed enrichment of indispensable genes in our electrophile-sensitive cysteines dataset relative to all protein-coding genes or protein-coding genes containing at least one cysteine. No difference in the distribution of phenotype scores was observed between the low-, medium- and high-electrophile-sensitivity groups. Phenotype scoring identified a focused group of 75 highly electrophile-sensitive cysteines in 56 essential genes (Supplementary Table 3). Phylogenetic analysis of these targets indicated varying degrees of cysteine conservation across different protein classes and eukaryotes (Fig. 1e). Interestingly, several sites appeared to be widely conserved in clinically important pathogens yet absent in the human host, suggesting the potential for these cysteines to be selectively targeted with cysteine-directed drugs.

## CORe platform rationale and design

**Fig. 2:**
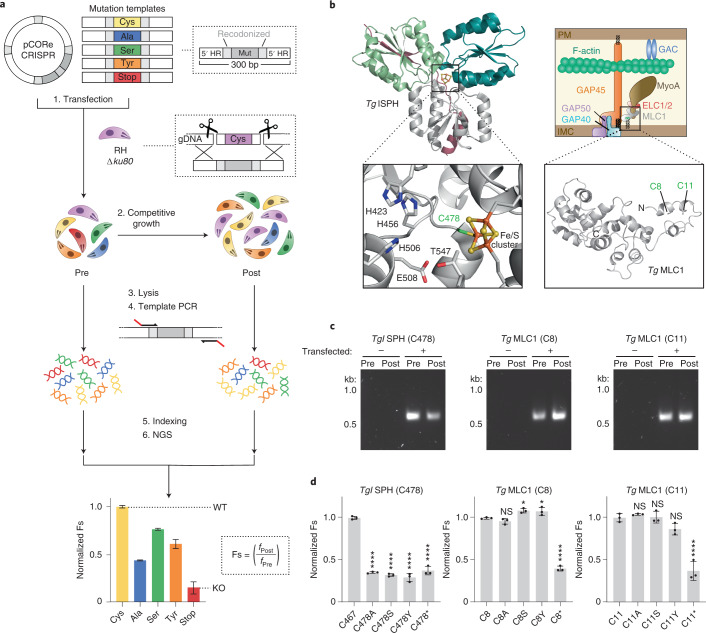
CORe discriminates between fitness-conferring and non-fitness-conferring chemically reactive sites. **a**, Workflow of CORe for functional interrogation of highly electrophile-sensitive cysteines in *T. gondii*. A single pCORe CRISPR plasmid is co-transfected into *T. gondii* parasites with a panel of linear double-stranded donor templates that encode different codon switches (a recodonized cysteine codon, alanine, serine, tyrosine and stop codon). Each plasmid encodes Cas9 nuclease and two gRNA cassettes that direct Cas9 to induce DSBs at sites 5′ and 3′ of a target cysteine codon. This promotes integration of templates at the excised genomic locus via HDR, substituting the endogenous cysteine for a given mutation. To increase the efficiency of HDR, a cell line deficient in NHEJ-based DNA repair is used (RHΔ*ku80*) (ref. ^56^). Genomic DNA from the transfected parasite population is extracted before (‘Pre’) and after (‘Post’) competitive lytic growth. For each timepoint, specific amplicons are generated by targeting primers to regions of recodonized sequence within the templates. The abundance of each mutation is quantified by NGS. The read frequency of each mutant in ‘Post’ (*f*_Post_) is normalized to ‘Pre’ (*f*_Pre_) to determine Fs that reflect the viability of parasites following amino-acid substitution. Fs values for the amino-acid substitutions are compared against the synonymous recodonized cysteine (WT) and stop codon (KO) mutations to identify deleterious mutations (that is, functional cysteines). **b**, Structural models of CORe targets *Tg*ISPH (left) and *Tg*MLC1 (right). Insets show the positions of their associated target cysteines. IMC, inner membrane complex; PM, plasma membrane; GAC, glideosome-associated connector. **c**, Amplicons generated following mutation of *Tg*ISPH (C478) and *Tg*MLC1 (C8/C11). Agarose gel shown is representative of three independent experiments. **d**, Histograms showing Fs values for cysteine mutants of *Tg*ISPH (C478) and *Tg*MLC1 (C8/C11), normalized to the recodonized cysteine control. Data represent mean ± s.d. values for three independent experiments (*n* = 3). Statistical significance for each mutant was compared against the recodonized cysteine control by one-way ANOVA with Dunnett’s correction for multiple comparisons (****P < 0.0001; *P < 0.05; NS, no significance, *P* > 0.05). Source data

To systematically interrogate cysteines identified by isoTOP-ABPP, we designed a methodology to prioritize individual sites on the basis of demonstrated contribution to protein function in cells. We refer to our approach as CORe (Fig. 2a). The underlying principle of CORe is that for essential genes there is a direct relationship between the molecular function of the encoded protein and cellular fitness; mutations that perturb protein function will similarly impact cellular fitness. By comparing the fitness of wild-type (WT) and cysteine mutants for a specific electrophile-sensitive cysteine-containing gene product, the functional contribution of the target residue can be assessed within the sequence context of the protein. This is achieved through site-specific integration of different mutations using a CRISPR–Cas9-based homology-directed repair (HDR) strategy, and subsequent quantitative comparison of the fitness of the resulting reactive site mutant(s) with WT and KO controls. Highly electrophile-sensitive cysteines were selected as targets for their higher intrinsic nucleophilicity to covalent ligands rather than any presumed or predicted functionality. This is important for downstream fragment-based lead discovery campaigns, which typically use warheads with lower reactivity than IAA. For functional interrogation of highly electrophile-sensitive cysteines in *T. gondii*, we selected five mutation types; a recodonized cysteine (synonymous replacement of the target cysteine; WT), a stop codon (for disruption of the target gene; KO^26^) and three distinct amino-acid substitutions: alanine, serine or tyrosine. While alanine and serine are commonly used in mutagenesis studies, tyrosine was included to probe sites that could participate in protein–protein interactions (PPIs). Meta-analysis of PPI mutation datasets obtained from cancer studies^27^ revealed that tyrosine is the most frequent cysteine substitution that causes destabilization at PPI interfaces (Supplementary Table 6). We reasoned that a tyrosine mutation might identify cysteine-dependent PPI hotspots, while acknowledging the caveat that a large aromatic substitution may affect protein function via folding defects. Details on the optimization of CORe are provided in Methods.

## CORe functionally prioritizes cysteine targets in live cells

**Extended Data Fig. 2:**
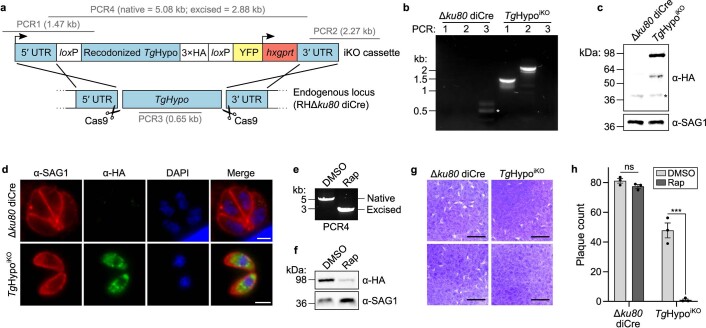
*Tg*Hypo is indispensable for *T. gondii in vitro*. **a**, Schematic of the CRISPR-based HDR strategy used for generating a *Tg*Hypo inducible knockout line (*Tg*Hypo^iKO^) using the diCre system. The predicted sizes of the PCR amplicons used for validating genomic integration and excision of loxP-flanked gene constructs are annotated. **b**, PCR products confirming correct integration of the floxed *Tg*Hypo construct at the 5′ and 3′ UTRs, and loss of the wildtype *Tg*Hypo at its endogenous locus. Agarose gel shown is representative of two independent experiments. **c**, Western blot showing expression of the 3×HA-tagged *Tg*Hypo construct in *Tg*Hypo^iKO^ parasites using an α-HA antibody; equal protein loading was verified using an α-SAG1 antibody. Image shown is representative of two independent experiments. **d**, Immunofluorescence micrographs of *Tg*Hypo^iKO^ parasites following staining with α-HA antibodies, showing correct cytosolic localization of the *Tg*Hypo-3×HA construct. SAG1 and DAPI were used as parasite surface and nuclear markers, respectively. Scale bar = 3 μm. Micrographs are representative of images captured from multiple fields of view across two independent experiments. **e**,**f**, Analytical PCR (**e**) and western blot (**f**) showing excision and degradation of the floxed *Tg*Hypo-3×HA construct in *Tg*Hypo^iKO^ parasites upon rapamycin treatment. Protein loading was assessed using an α-SAG1 antibody. Image scans are representative of results from two independent experiments. **g**, Representative images of plaques formed on HFF monolayers by the indicated strains in the presence of rapamycin or DMSO. Scale bar = 0.5 cm. **h**. Plaque counts for each strain determined from (**g**) showing loss of plaquing capacity in *Tg*Hypo^iKO^ parasites upon rapamycin treatment. Data are presented as mean ± SEM across three independent experiments (n = 3), with each point representing mean counts from three technical replicates. Statistical significance was determined by one-way analysis of variance (***p < 0.001; ns = no significance, *p* > 0.05). *p* values: Δ*ku80* diCre DMSO vs Rap, 0.13; *Tg*Hypo^iKO^ DMSO vs Rap, 0.0007. Source data

We trialled CORe against electrophile-sensitive cysteines in two targets: *Tg*ISPH (TGGT1_227420) and *Tg*MLC1 (TGGT1_257680) (Fig. 2b). ISPH (also known as ‘LytB’) is an oxidoreductase essential for isoprenoid biosynthesis and an established anti-microbial drug target^28,29^. The electrophile-sensitive cysteine identified in *Tg*ISPH contributes to an iron–sulfur cluster that is required for enzyme catalysis, and therefore any substitutions at this site are expected to be deleterious. *Tg*MLC1 is part of the glideosome complex required for parasite motility and host-cell invasion^30^ and contains two N-terminal electrophile-sensitive cysteines that are known to be *S*-acylated^31,32^ but do not contribute *Tg*MLC1 function^33^. We predicted that substitutions at these residues would not affect parasite fitness. We applied CORe to these targets; integration-specific amplicons were successfully generated (Fig. 2c), and next-generation sequencing (NGS) analysis confirmed our expectations with high biological reproducibility. Any substitution of the *Tg*ISPH-associated cysteine negatively impacted parasite fitness, with all three mutations being analogous to disruption of the gene following integration of the stop codon (Fig. 2d). In agreement with published data, the cysteines on *Tg*MLC1 were permissive to all mutations, indicating that these residues (and their post-translational modification) do not contribute to the essential component of this protein’s function (Fig. 2d). We next benchmarked a standard genetic analysis workflow against which CORe could be compared. For this purpose, we selected a hyperreactive cysteine associated with a hypothetical protein (TGGT1_258070). We generated an inducible KO (iKO) line using the DiCre system (RH *Tg*Hypo^iKO^) (Extended Data Fig. 2a), and confirmed the expected genomic re-arrangement by polymerase chain reaction (PCR), expression of the protein by western blot, and localization by immunofluorescence microscopy (Extended Data Fig. 2b–d). Treatment of RH *Tg*Hypo^iKO^ parasites with rapamycin resulted in efficient gene excision and protein degradation (Extended Data Fig. 2e,f), and in agreement with the gene’s phenotype score (−5.24) plaque assay confirmed that KO parasites were not viable (Extended Data Fig. 2g,h). We then sought to assess the contribution of the reactive cysteine to protein function by genetic complementation in the genetic background of this iKO. We were unable to complement for the loss of this gene, precluding functional interrogation of the associated cysteine. Supplementary Fig. 1 summarizes the comparative timeline for CORe with this standard approach.

**Fig. 3:**
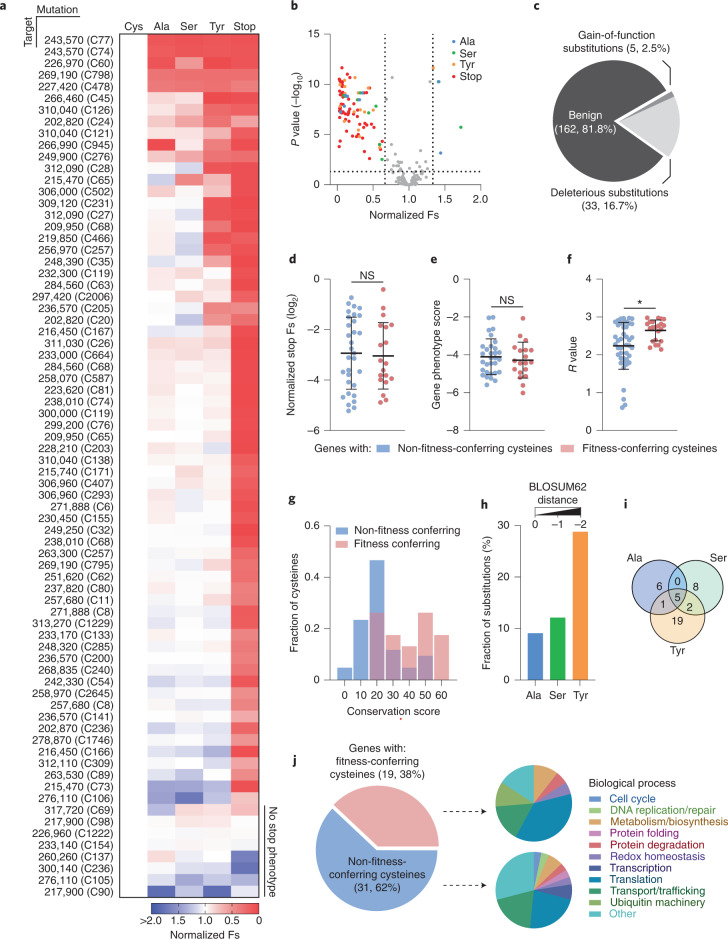
CORe prioritizes apicomplexan protein translation as a target for covalent inhibition. **a**, Heat map showing normalized Fs values for all target cysteines and mutation types ordered by the mutation sensitivity of the cysteines (high to low, top to bottom). **b**, Volcano plot showing the normalized Fs values of each cysteine mutation and statistical significance against the recodonized cysteine control as determined by one-way ANOVA. Data represent mean Fs values for three independent experiments (*n* = 3). Significant mutations (*P* < 0.05) with mean Fs values <0.66 and >1.33 represent deleterious and gain of function, respectively, and are coloured. Only cysteines (66/74) featuring a deleterious stop codon mutation are shown and used in subsequent analyses. **c**, Proportion of amino-acid substitutions causing deleterious or gain-of-function phenotypes. **d**,**e**, Distribution of normalized stop codon Fs values (**d**) and phenotype scores (**e**) between genes containing at least one (*n* = 19) or no fitness-conferring cysteines (*n* = 31), and isoTOP-ABPP *R* values (**f**) of the fitness conferring (*n* = 23) or non-fitness conferring cysteines (*n* = 43) themselves. Bars represent mean ± s.d. Statistical differences between group means were assessed by two-tailed Student’s *t*-tests (*P < 0.05; NS, no significance, *P* > 0.05). *P* values: stop codon Fs values, 0.6702; phenotype scores, 0.3498; *R* values, 0.0150. **g**, Frequency distribution of conservation scores assigned to fitness-conferring and non-fitness-conferring cysteines across 20 eukaryotic organisms; higher scores indicate wider conservation across the analysed species. **h**, Fraction of deleterious amino-acid substitutions for each mutation type. The BLOSUM62 distance scores for each substitution are annotated and organized by increasing distance from the native cysteine residue (left to right). **i**, Overlap of cysteines with deleterious alanine, serine and/or tyrosine substitutions. **j**, Proportion and functional annotations of proteins containing fitness-conferring and non-fitness-conferring cysteines. Source data

**Extended Data Fig. 3:**
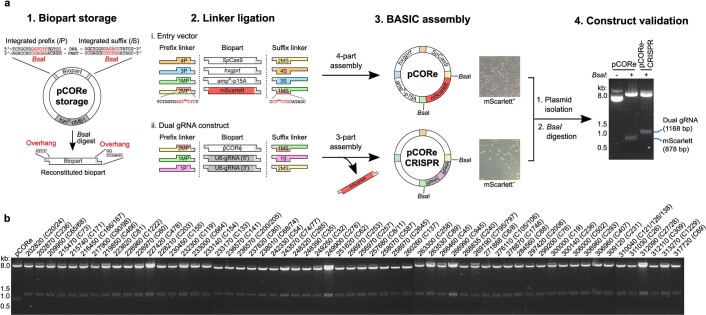
Biopart Assembly Standard for Idempotent Cloning (BASIC) enables modular, high-throughput assembly of CORe CRISPR plasmids. **a**, BASIC strategy used for plasmid construction. (1) The BASIC physical DNA standard. Functional DNA bioparts are flanked by *i*P and *i*S sequences, each containing a *Bsa*I restriction site (red). In CORe, BASIC parts are released from kanamycin-resistant storage plasmids (pCORe storage) by *Bsa*I digestion, enabling the ligation of oligonucleotide linkers for subsequent vector assembly via the BASIC workflow^53^. (2,3) Assembly strategy for the CORe entry vector (‘pCORe’; i) and final dual gRNA constructs (‘pCORe CRISPR’; ii). pCORe is generated through the ordered assembly of four bioparts: *Sp*Cas9, *hxgprt*, amp^R^-p15A and mScarlett. Bacterial transformants of pCORe exhibit a pink phenotype due to expression of the mScarlett fluorophore. The methylated cytosines uniquely present in the linkers flanking mScarlett prevent digestion of the linker during the assembly process and reconstitutes pCORe (*Sp*Cas9-*hxgprt*-amp^R^-p15A) for a second round of assembly. The pCORe biopart is then subject to a 3-part assembly reaction with two gRNA parts, replacing the mScarlett cassette and generating pCORe CRISPR. Transformants of pCORe CRISPR appear non-fluorescent due to the loss of the mScarlett marker, enabling rapid selection of successful assemblies. (4) Following plasmid isolation, successful insertion of the two gRNA parts is verified by differential size analysis of fragments upon *Bsa*I digestion. **b**, *Bsa*I verification of a 59-member pCORe CRISPR library targeting 74 hyperreactive cysteines of *T. gondii*; successful gRNA insertion was achieved for all selected clones. Image scans shown represent a single experiment.

**Extended Data Fig. 4:**
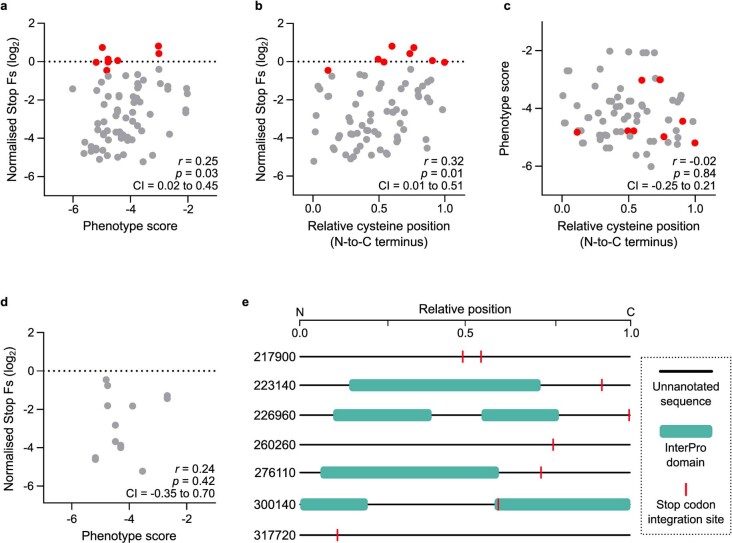
CORe stop codon mutant fitness does not correlate with cysteine position or gene phenotype scores. **a**,**b**. Correlation of normalised mean stop codon Fs values (**a**) and phenotype scores (**b**) against the relative position of the mutagenized cysteine in the associated protein sequence. While no overall correlation is observed, targets without a statistically significant stop codon phenotype (colored red) generally cluster toward the C terminus. **c**,**d**. Comparison of statistically significant stop codon mutant phenotypes against published phenotype scores^25^ for all interrogated genes (**c**) or interrogated genes with cysteines close to the protein N-terminus (relative cysteine position ≤ 0.2, **d**); no overall relationship is observed. Annotated *r* values indicate the degree of correlation between datasets being compared following a two-tailed Pearson’s correlation analysis. Annotated *p* and CI values represent the statistical significance and 95% confidence intervals of the correlation analysis, respectively. **e**. Relative positions of functional domains (predicted by InterPro; www.ebi.ac.uk/interpro) and mutagenized cysteines across the primary sequences of targets undisrupted by stop codons. The numerical *Toxo*DB gene IDs for each target are listed. Source data

We applied CORe to our complete set of highly electrophile-sensitive cysteine-containing essential genes. Construction of 59 CRISPR plasmids was accomplished in 5 days using a linker-based DNA assembly strategy (Extended Data Fig. 3a,b), followed by parasite transfection and competitive lytic growth (8 days), integration-specific amplicon production (achieving 100% coverage for our target cysteines; Supplementary Fig. 2), NGS library construction (7 days) and Illumina NextSeq processing (7 days). The entire CORe workflow took approximately 1 month to complete for 74 electrophile-sensitive cysteine targets, with the final dataset indicating exceptional reproducibility across independent biological replicates. These data are summarized in Fig. 3a and Supplementary Fig. 3. For ~90% of the target cysteines (66/74), the integration of the premature stop codon resulted a significant (*P* < 0.05) reduction in parasite fitness (Fig. 3b). Interestingly, the relative magnitude of the effect of integrating the stop codon did not correlate with published gene phenotype scores (Extended Data Fig. 4a,d), and no deleterious growth phenotype was detected for stop codon mutants in eight targets. This may reflect the proximity of these mutagenized cysteine to the protein C-terminus as these proteins could retain functional domains. Despite a lack of overall correlation between cysteine position and stop codon disruption or phenotype score (Extended Data Fig. 4b,c), several of these are positioned C-terminally to predicted domains (Extended Data Fig. 4e).

**Extended Data Fig. 5:**
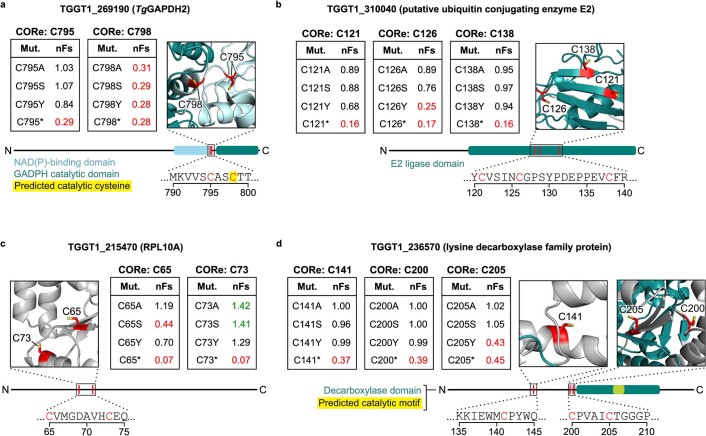
Electrophile-sensitive cysteines within individual proteins have diverse mutational tolerances. **a-d**, CORe mutagenesis profiles for target cysteines in TGGT1_269190 (**a**), TGGT1_310040 (**b**), TGGT1_215470 (**c**) and TGGT1_236570 (**d**). Inset tables show the average normalized Fs (nFs) for each cysteine upon alanine, serine, tyrosine and stop codon substitution; deleterious and gain-of-function mutations are colored in red and green, respectively. The relative positions of the mutagenized cysteines across the associated protein sequence are highlighted red in the domain architecture schematic alongside domains and motifs predicted by InterPro (www.ebi.ac.uk/interpro). The sequence context of these sites and their locations within the tertiary protein structure (predicted using AlphaFold 2.0^35^) are highlighted in the inset.

Analysis capturing aspects of both the magnitude and statistical significance of the effect of each individual substitution identified robustly fitness conferring cysteines, prioritizing target sites according to their contribution protein function in live cells (Fig. 3b and Supplementary Table 7). The majority of substitutions were benign (~83%, 184/222), with only a small fraction of the highly electrophile-sensitive cysteines measurably contributing to the function of the protein (~17%, 38/222) (Fig. 3c). Unexpectedly, CORe identified gain- as well as loss-of-function mutations. For several targets (for example, *Tg*GAPDH2), we also recognized that individual cysteines can show different mutational tolerances (Extended Data Fig. 5), indicating that CORe can effectively discriminate between functional and non-functional sites within a single protein. Illustrating the challenge of selecting targets in the absence of an approach such as CORe, there was no association between the Fs of a given cysteine and the effect of stop codon integration (Fig. 3d), the phenotype score of the associated gene (Fig. 3e) or increased electrophile sensitivity of the cysteine itself (Fig. 3f). The challenge of target selection is highlighted by the cysteine chosen for validation via the standard genetic workflow (Extended Data Fig. 2). For this hypothetical protein, CORe indicated that the reactive cysteine does not contribute to protein function (Fig. 3a and Supplementary Fig. 3). Addressing the relationship between cysteine ‘essentiality’ and function, we compared the extent of conservation for fitness-conferring and non-fitness-conferring cysteines according to ‘conservation scores’ (Fig. 3g and Supplementary Table 5). While non-fitness-conferring cysteines appeared to be normally distributed across the analysed species, fitness-conferring cysteines displayed a bimodal distribution with higher scores. This indicated that conservation should not be taken as the sole predictor of function.

**Extended Data Fig. 6:**
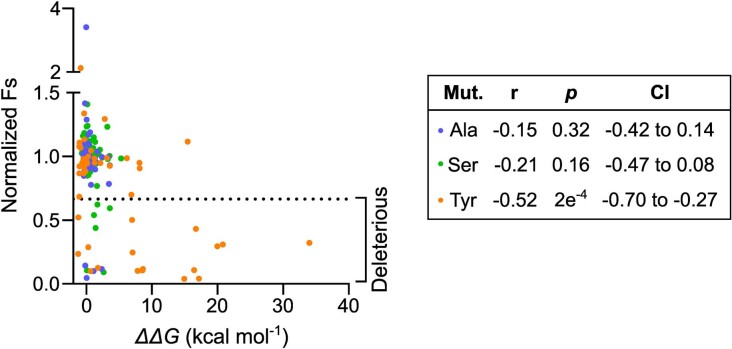
Protein folding stability is not substantially impacted by specific substitution type(s). Protein structures for 35 genes interrogated by CORe were obtained from the RCSB PDB, or from high-confidence models predicted by Phyre2 or AlphaFold 2.0^35,36^. The impact of cysteine substitutions (alanine, serine and tyrosine) on the free energy of folding (ΔΔ*G*) was assessed using the PositionScan function of FoldX^37^, where negative and positive ΔΔ*G* values reflect an increase and decrease in folding stability relative to the wildtype model, respectively. Scatter plot shows the relationship between ΔΔ*G* and the normalized Fs value for the associated cysteine mutation. Annotated *r* values indicate the degree of correlation between ΔΔ*G* and Fs for each mutation type following a two-tailed Pearson’s correlation analysis. The statistical significance (*p*) and 95% confidence (CI) values are noted. While a subset of tyrosine substitutions have a destabilizing effect, there is no overall correlation. Source data

Integration of three different amino-acid substitutions enabled deeper interrogation of each site, and an increased appreciation of functionally disruptive biochemistry (Fig. 3h). In agreement with anticipated evolutionary mutational tolerance, the greater the BLOSUM62 matrix distance^34^ between the individual mutation and cysteine, the more likely the mutation affected the function of the associated protein. This supports a degree of functional buffering or resistance against gradual evolutionary change of protein function as a result of changes in protein sequence, with a range of tolerance observed for each individual cysteine (Fig. 3i). We recognized that the functional annotations provided by CORe might encompass impacts upon protein folding and stability. To test this, we obtained or predicted high-confidence structures for 35/56 genes using the Protein Data Bank, AlphaFold^35^ or Phyre2^36^, and used FoldX^37^ to predict the change in Gibbs free energy (∆∆*G*) resulting from the cysteine substitutions. These ∆∆*G* values were then compared with CORe Fs values (Extended Data Fig. 6). While the more radical tyrosine substitution was predicted to have a greater impact upon protein stability for a subset of targets, there was no clear correlation between CORe Fs, ∆∆*G* and a particular substitution type.

**Extended Data Fig. 7:**
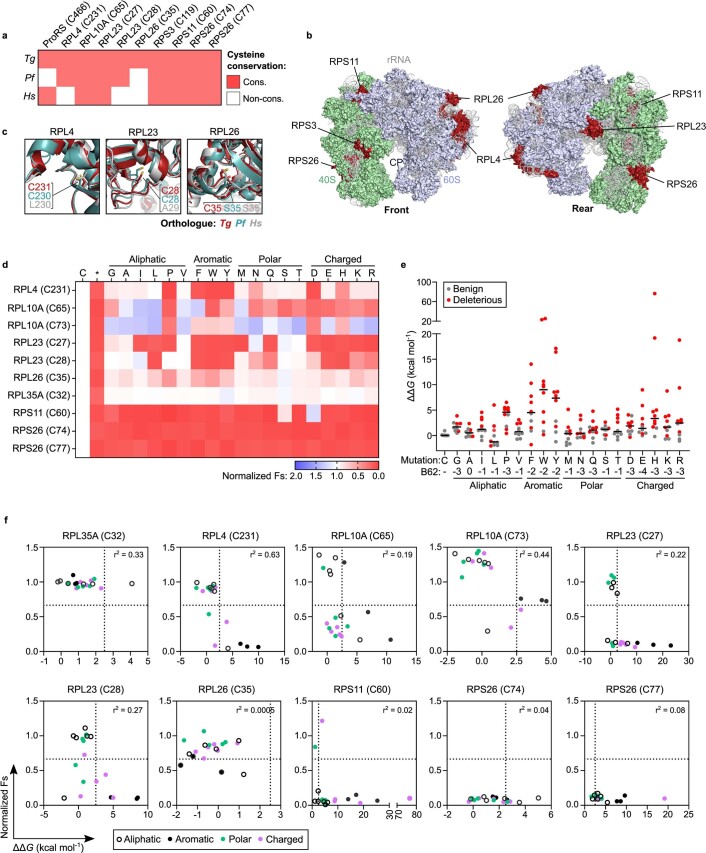
Saturation mutagenesis of electrophile-sensitive cysteines in ribosomal proteins in T. gondii has diverse impact on parasite fitness and protein folding stability. **a**, Conservation of fitness-conferring cysteines identified in translation-associated proteins of *T. gondii* (*Tg*) in orthologues of *P. falciparum* (*Pf*) and *H. sapiens* (*Hs*). **b**, Front (left) and rear (right) views of the cytoplasmic *Tg* 80 S ribosome (PDB 5XXU/5XXB). Ribosomal subunits, RNA and proteins containing fitness-conferring cysteines are colored and annotated. **c**, Structural alignment of selected ribosomal proteins with orthologues in the *Pf* (6OKK/3J79) and *Hs* (4UG0) 80 S ribosomes. The cysteines and their positional equivalents in *Pf* and *Hs* are represented in stick form and annotated. **d**, Heatmap displaying normalized Fs values following saturation mutagenesis of 10 electrophile-sensitive cysteines in ribosomal proteins by CORe. Amino acid substitutions are grouped by the biochemical properties of the associated side chain. Data represents mean values from 3 independent experiments (n = 3). **e**, Effects of different amino acid substitutions from **d** on predicted protein folding stability. Changes in the free energy of folding (ΔΔ*G*) was assessed for each substitution using FoldX^37^, where negative and positive ΔΔ*G* values reflect increasing and decreasing stability relative to the wildtype protein, respectively. Data mean ΔΔ*G* values for each substitution type across 10 distinct targets (n = 10). The BLOSUM62 (B62) distances for each amino acid from the wildtype cysteine residue are annotated. **f**, Scatter plots comparing Fs and ΔΔ*G* values for 19 amino acid substitutions in 10 electrophile-sensitive cysteines. Substitutions are colored according to their side chain chemistries; horizontal and vertical lines reflect thresholds for deleterious (0.66 > Fs) and destabilizing (2.5 < kcal/mol) substitutions, respectively. Source data

**Extended Data Fig. 8:**
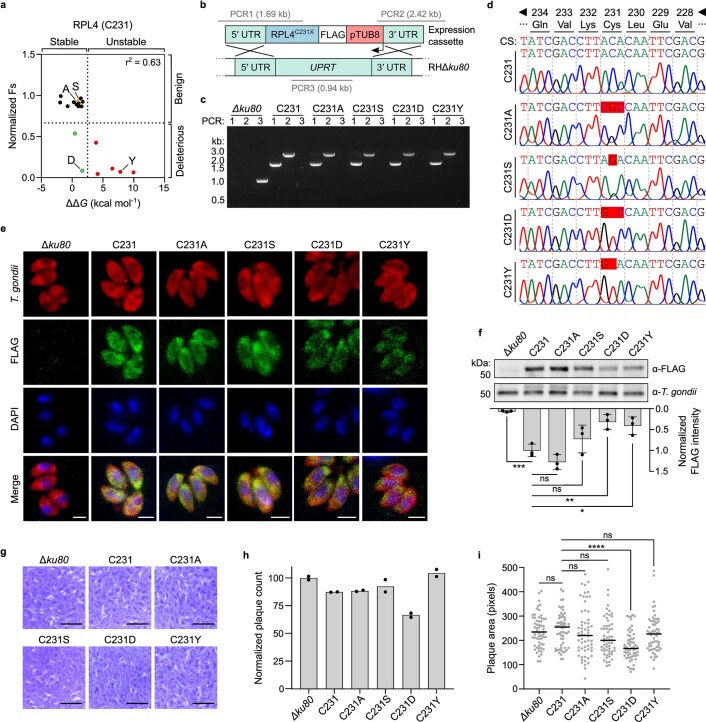
Identification of physiologically relevant cysteine substitutions in RPL4. **a**, Normalized Fs values for amino acid mutants of RPL4 (C231) plotted their predicted folding free energies (ΔΔ*G*), as determined by CORe and FoldX^37^, respectively. **b**, Genetic engineering strategy used to generate *T. gondii* parasite lines expressing FLAG-tagged C231 mutants of RPL4 at the UPRT locus (RHΔ*ku80*^RPL4(C231X)^). The sizes of the PCR amplicons used to assess integration of the expression constructs are annotated. **c**, PCR products confirming correct integration of the wildtype and C231A/S/D/Y RPL4 expression constructs at the UPRT 5′ and 3′ UTRs, and disruption of endogenous UPRT locus. Image shown is representative of results from two independent experiments. **d**, Sequence chromatograms of the integrated RPL4 constructs confirming appropriate mutagenesis of C231. **e**, Immunofluorescence micrographs of RHΔ*ku80*^RPL4(C231X)^ parasites following staining with an α-FLAG antibody confirming expression and the expected cytosolic localisation of all RPL4 constructs. Parasite surfaces and nuclei were stained with an α-*T. gondii* antibody and DAPI, respectively. Scale bar = 3 µm. Micrographs are representative of images captured across three technical replicates of a single experiment. **f**, Western blot confirming expression of the RPL4 constructs in RHΔ*ku80*^RPL4(C231X)^ parasites using an α-FLAG antibody; protein loading was assessed with an α-*T. gondii* antibody (top). The FLAG staining intensities for each mutant were quantified, normalized and statistically compared to the RHΔ*ku80*^RPL4(C231)^ control (bottom) by one-way analysis of variance (***p < 0.001; **p < 0.01; *p < 0.05; ns = no significance, *p* > 0.05). Data represents mean ±s.d. intensity from three independent experiments (n = 3), revealing notably reduced expression for deleterious C231D and C231Y variants. *p* values: C231 vs Δ*ku80*, 0.0305; C231 vs C231A, 0.3557; C231 vs C231S, 0.3625; C231 vs C231D, 0.0055; C231 vs C231Y, 0.0147. **g**, Representative images of plaques formed on HFF monolayers by the indicated strains. Scale bar = 0.5 cm. **h**, Plaque counts (**h**) and area (**i**) for each strain depicted in **g**, showing a reduction in plaquing ability in the RPL4 C231D mutants. Data represents mean counts from two independent experiments (n = 2), with each point indicating the average counts from three technical replicates. For plaque area, the size of sixty randomly selected plaques (n = 60) are shown, with the mean area indicated by black bars. Differences in plaque area of each mutant against the RHΔ*ku80*^RPL4(C231)^ control line were statistically assessed by one-way analysis of variance (****p < 0.0001; ns, *p* > 0.05). *p* values: C231 vs Δ*ku80*, 0.9984; C231 vs C231A, 0.9784; C231 vs C231S, 0.9784; C231 vs C231D, < 0.0001; C231 vs C231Y, 0.8502. Source data

To improve our chances of finding an individual druggable target, we initially sought to identify broader biological processes with sensitivity to cysteine-reactive covalent small molecules. We performed an enrichment analysis of functional annotations assigned to proteins containing fitness-conferring or non-fitness-conferring cysteines (as defined by CORe) (Fig. 3j). The breakdown for the two groups was distinct, with ‘translation’ annotation enriched in genes containing fitness-conferring cysteines. We undertook an in-depth analysis of CORe-prioritized electrophile-sensitive cysteines present on proteins associated with translation. The majority of these sites (9/10) were encoded in proteins decorating the surface of the cytoplasmic 80S ribosome (Extended Data Fig. 7a–c). To further understand the mutational tolerance of these residues, we applied CORe for site-saturation mutagenesis of ten ribosomal cysteines. As expected, CORe identified a broad spectrum of fitness changes (Extended Data Fig. 7d, Supplementary Fig. 4 and Supplementary Table 8). This included residues tolerant to all substitutions, as well as sites intolerant to any substitution presumably where the functional contribution of the residue is dependent on the cysteine thiol. FoldX analysis indicated that, while aromatic substitutions had the greatest impact on protein folding stability (Extended Data Fig. 7e), there was no consistency in the effect of deleterious substitutions upon ∆∆*G* across targets (Extended Data Fig. 7f). We focused on RPL4(C231) to validate these data as the mutational profile for this site suggested that specific amino-acid side chains were not tolerated. Dedicated ribosome assembly chaperones Kap104 and Acl4 have been documented to sequester Tom1-dependent polyubiquitination sites present on the surface of yeast RPL4, and thereby protect unassembled RPL4 from proteasomal degradation^38^. We hypothesized that, should these interactions be conserved in *T. gondii*, deleterious RPL4(C231) substitutions may not be tolerated owing to a disruptive effect on these PPIs leading to increased cellular degradation of RPL4. Our saturated substitution dataset and FoldX analysis directed our selection of conservative benign (C231A/S) and non-conservative substitutions found to be fitness conferring but with different predicted effects on ∆∆*G* (C231D/Y) (Extended Data Fig. 8a). We generated parasite lines ectopically overexpressing a second copy of RPL4 and C231 mutants, confirming the genomic integration, expression and expected localization of all constructs (Extended Data Fig. 8b–e). Consistent with the CORe readout, the synonymous cysteine and C231A/S mutants had similar protein expression levels and growth phenotypes (Extended Data Fig. 8f–i). In agreement with their deleterious fitness effects, expression levels of the C231Y/D mutants were notably lower. While overexpression of the C231Y mutant did not impact parasite lytic growth, a reduction in plaque area was observed for the C231D mutant (Extended Data Fig. 8g–i). Considering the relatively low predicted impact of C231D on protein folding (Extended Data Fig. 8a), this suggests that the effect of this substitution upon protein levels is not solely due to changes in stability.

## Covalent inhibition of malaria parasite protein translation

**Fig. 4:**
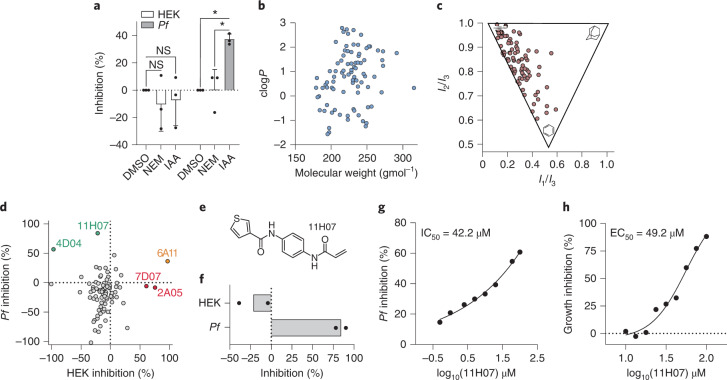
The apicomplexan translation machinery is selectively inhibited by thiol-reactive small molecules. **a**, Mean ± s.d. inhibition of *P. falciparum* (*Pf*) and HEK293 (HEK) IVT with 100 µM IAA or *N*-ethylmaleimide (NEM). Translational output was measured from cell lysates using a luciferase-based IVT assay^41^. DMSO was used as a vehicle control. Statistical significance was determined from three independent experiments (*n* = 3) by two-way ANOVA with Šidák’s correction for multiple comparisons (*P < 0.05; NS, no significance, *P* > 0.05). HEK *P* values: DMSO versus NEM, 0.7124; DMSO versus IAA, 0.8754. *Pf* P values: DMSO versus NEM, 0.0114; DMSO versus IAA, 0.0122. **b**, Molecular weight versus the clog*P* value of 88 acrylamide-containing fragments. **c**, Normalized PMI ratios of the acrylamide fragment library. Ratios are plotted in a triangular graph to depict the molecular shape diversity, where the vertices represent a perfect rod (*x* = 0, *y* = 1), disc (*x* = 0.5, *y* = 0.5) and sphere (*x* = 1, *y* = 1). **d**, Mean percentage inhibition of HEK293 and *Pf* IVT following treatment of cell lysates with 100 µM of each acrylamide fragment. Representative compounds inhibiting *Pf*, HEK293 or both lysates are annotated green, red and orange, respectively. **e**, Chemical structure of hit compound, 11H07. **f**, Expanded IVT profile for 11H07 from **d**. **g**,**h**, Concentration-dependent inhibition of *Pf* IVT (**g**) and growth (**h**) with 11H07. Data in **d** and **f**–**h** represent two independent experiments (*n* = 2), each with three technical replicates. Source data

Malaria remains a cause of substantial mortality and morbidity worldwide, and owing to existing and emergent drug resistance there is a constant demand for new therapeutic targets and modalities. Our cross-disciplinary pipeline prioritized protein translation for further target-based screening. Translation has a track record as a therapeutic target, including the malaria parasite, *P. falciparum*^39,40^, but has not previously been targeted for covalent inhibition. The majority of fitness-conferring translation-associated cysteines in *T. gondii* were conserved in *P. falciparum*. Interestingly, not all were conserved in humans, indicating the possibility of parasite-specific functions that could be therapeutically targeted (Extended Data Fig. 7a,c). An established assay for in vitro translation (IVT) is not currently available for *T. gondii*. With that in mind, we took advantage of an IVT assay recently established for *P. falciparum*^41^ to test the sensitivity of both *P. falciparum* and human translation to covalent inhibition with the promiscuous cysteine alkylating molecule, IAA. Parasite, but not human, translation, was sensitive to inhibition (Fig. 4a). Interestingly, no inhibitory effect was observed with another cysteine alkylator, *N*-ethylmaleimide, suggesting IAA-mediated translation inhibition was not solely due to indirect modification of non-protein thiols (for example, glutathione). Encouraged by these data, we assembled a focused library of 88 structurally diverse cysteine-reactive fragments bearing acrylamide warheads (Fig. 4b,c and Supplementary Tables 9 and 10). Screening this library against *P. falciparum* and human IVT identified a lead parasite-selective molecule, 11H07, with a translation half-maximal inhibitory concentration (IC_50_) of 42 (±9.8) µM (Fig. 4d–g and Supplementary Table 11). Our lead fragment also inhibited the growth of *P. falciparum* asexual blood stages in vitro, with a half-maximal effective concentration (EC_50_) of 49 (±1.0) µM. Combined, these data validated cysteine-targeted inhibition of protein translation in the malaria parasite as a new potential therapeutic modality for this biological process, paving the way for optimization and target deconvolution of our lead compound.

## Discussion

In recent years the scope, scale and speed with which chemically reactive amino acids can be profiled has accelerated dramatically; chemoselective probes are now available for cysteine^4^, serine^42^, lysine^5^, methionine^6^, tyrosine^7^, aspartate/glutamate^9,43^, tryptophan^44^ and histidine^45^. Supporting this, advances in MS have greatly expanded the number and rate at which individual reactive sites can be profiled^46,47^, and subsequently exploited by electrophilic drug hunters. CORe provides a technology bridge between unbiased proteomic profiling of chemically reactive sites and protein sequence–function relationships, and will be a valuable tool for proteome engineers alongside other methods, including MAGE, CRMAGE and CREATE. CORe provides a simple strategy to interrogate any individual amino acid, directly assessing its contribution to protein function, which we anticipate will prove as useful as alanine scanning in traditional protein structure–function studies. CORe is similar to other saturation mutagenesis approaches, but whereas these methods mutate entire domains to saturation, we focus our substitution analyses on chemically reactive amino acids on protein surfaces. This is highly beneficial when hunting new targetable sites, as electrophile-sensitive residues are, by definition, chemically accessible. Surprisingly, we observed no clear association between the relative reactivity (as measured by isoTOP-ABPP *R* values) of the cysteines interrogated by CORe and their likelihood of being fitness conferring in the parasite (Fig. 3f). Our data indicate that, contrary to expectation from other published work, cysteine electrophile sensitivity does not predict functionality in *T. gondii*. Extended consideration of our data is presented in the supplementary discussion. While *T. gondii* was used for proof-of-concept studies with electrophile-sensitive cysteines, CORe is amino acid agnostic, with one exciting future application being the systematic profiling of all post-translationally modified proteins of a given class, such as sites of phosphorylation or *N*-myristoylation. Many advances in the target identification and validation sphere are currently achieved retrospectively following identification of a suitable ligand. In contrast, global amino-acid chemical reactivity profiling combined with CORe supports prospective strategies in target identification and validation campaigns. Our approach enables the critical concept of prioritization to be used to promote protein targets and targetable biological processes into screening platforms where an identified prospectively druggable site is already proven to contribute to protein function in intact cell systems. As such, combined with chemical reactivity profiling, CORe has the potential to focus drug discovery pipelines on functional sites on identified targets, accelerating the discovery of targets and next-generation small-molecule therapeutics. The translation of our findings to the related malaria parasite *P. falciparum* provides the first evidence for this potential, with covalent inhibition of apicomplexan parasite translation apparatus being a tantalizing modality for new broad-spectrum anti-microbials.

## Methods

### General

Unless otherwise stated, all reagents were provided by Sigma. All primers/oligonucleotides and synthetic DNA used in this study are listed in Supplementary Tables 12 and 13, respectively. The IA-alkyne probe, Azo-L and Azo-H tags were synthesized as previously described^4,22^.

### Cell culture and parasite isolation

RH strain *T. gondii* tachyzoites were cultured by serial passage on confluent monolayers of human foreskin fibroblasts (HFFs; HFF-1 ATCC SCRC-1041). HFFs were grown at 37 °C and 5% CO_2_ in Dulbecco’s modified Eagle’s medium supplemented with 10% (v/v) heat-inactivated foetal bovine serum, 100 µg ml^−1^ penicillin/streptomycin and 2 mM l-glutamine. Unless otherwise stated, parasites were collected for assays or transfection via mechanical syringe lysis of heavily infected HFFs through a 25-gauge needle.

Highly synchronized 3D7 strain *P. falciparum* asexual parasites were cultured in RPMI-1640 medium supplemented with 0.5% (w/v) AlbuMAX II (Life Technologies), 50 μg ml^−1^ hypoxanthine, 25 μg l^−1^ gentamycin and 0.3 mg ml^−1^ l-glutamine. Parasites were routinely cultured at 37 °C and 5% CO_2_/3% O_2_ with 2% haematocrit blood (NHS UK Blood Transfusion Service). Medium was exchanged daily until the culture reached 10–20% parasitaemia with predominantly late trophozoites and early schizonts. Infected red blood cells (RBCs) were isolated by centrifugation (800*g*, 5 min) and lysed in RBC lysis buffer (45 mM HEPES pH 7.45, 100 mM potassium acetate, 1.5 mM magnesium acetate, 2 mM DTT and 0.075% (w/v) saponin) for 10 min at room temperature. The lysed RBCs were then centrifuged (2,800*g* and 4 °C, 10 min), and the resulting parasite pellet was suspended in cell lysis buffer (45 mM HEPES pH 7.45, 100 mM potassium acetate, 1.5 mM magnesium acetate and 2 mM DTT). This step was repeated until all RBC debris was removed.

HEK 293F cells were cultured in FreeStyle 293 Expression Medium (Life Technologies) at 37 °C and 5% CO_2_. Cells were collected at a density of ~2 × 10^6^ ml^−1^ by centrifugation (1,000*g* for 10 min at 4 °C) and washed once in cell lysis buffer supplemented with 20 U of human placental RNase inhibitor and cOmplete EDTA-free Protease Inhibitor Cocktail (Roche) before processing lysates.

All parasite and host cell strains were confirmed negative for the presence of *Mycoplasma* contamination by PCR.

### Plasmid design and construction

To construct pG140::*Tg*Hypo-3×HA, a recodonized *Tg*Hypo complementary DNA sequence fused to a C-terminal 3× HA tag was synthesized by GeneArt (Life Technologies). This fragment was cloned into the *Bam*HI and *Hind*III sites of a modified version of the parental plasmid p5RT70loxPKillerRedloxPYFP-HX^48^, in which the *TUB8* promoter had been deleted using the Q5 Site-Directed Mutagenesis Kit (NEB) protocol with primers P1/P2. Next, fragments encompassing the *TgHypo* 5′ or 3′ untranslated region (UTR) were PCR amplified from genomic DNA of RHdiCreΔ*ku80*Δ*hxgprt* parasites using primers P3/P4 and P5/P6, respectively. The 5′ UTR fragment was cloned into the *Nar*I site of the intermediate plasmid, followed by the 3′ UTR fragment at the *Sac*I site to generate pG140::*Tg*Hypo-3×HA.

To construct pSAG1::Cas9-U6::sg*Tg*Hypo(×2), Cas9 sgRNA sequences targeting the *Tg*Hypo 5′ or 3′ UTR were first selected using the Eukaryotic Pathogen gRNA Design Tool (EuPaGDT)^49^. Two single gRNA vectors containing either the 5′ UTR-targeting gRNA or the 3′ UTR-targeting gRNA were then generated using the pSAG1::Cas9-U6::sgUPRT plasmid as a backbone (Addgene #54467) (ref. ^50^). Here, the parental UPRT-targeting gRNA was replaced with either *Tg*Hypo gRNA using the Q5 Site-Directed Mutagenesis Kit protocol with primers P7/P9 (5′ gRNA) and P8/P9 (3′ gRNA). Next, a fragment encompassing the 5′ gRNA was PCR amplified using primers P10/P11 and Gibson cloned^51^ into the other *Kpn*I and *Xho*I-digested 3′ gRNA plasmid, generating pSAG1::Cas9-U6::sg*Tg*Hypo(×2).

To construct pUPRT::FLAG-RPL4, a recodonized RPL4 cDNA fragment with an N-terminal FLAG tag was cloned into the *Eco*RI and *Pac*I sites of the expression vector pTUB8-roGFP^52^. An expression cassette encompassing both the TUB8 promoter and FLAG-RPL4 cassette (pTUB8-FLAG-RPL4) was PCR amplified from the intermediate expression plasmid using primers P12/P13 and assembled with a UPRT-targeting backbone via Gibson assembly. To generate the pUPRT::FLAG-RPL4 mutant derivatives (pUPRT::FLAG-RPL4^C231A/S/D/Y^), point mutations were introduced into the recodonized RPL4 sequence in pUPRT::FLAG-RPL4 using the Q5 Site-Directed Mutagenesis Kit with primers P14/18 (C231A), P15/P18 (C231S), P16/P18 (C231D) or P17/P18 (C231Y).

All CORe plasmids were assembled by Biopart Assembly Standard for Indempotent Cloning (BASIC)^53^. To construct the pCORe recipient vector, three DNA parts (a Cas9 nuclease, *hxgprt* selectable marker and an mScarlett counterselection cassette) were generated with flanking BASIC Prefix and Suffix sequences. The Cas9 part was generated via PCR amplification of pCas9/Decoy (Addgene #80324) (ref. ^25^) using primers P19/P20. The mScarlett part was synthesized by Twist (www.twistbioscience.com). The *hxgprt* part was amplified from pTUB1:YFP-mAID-3HA, DHFR-TS:HXGPRT (Addgene #87259) (ref. ^54^) using primers P21/P22. Before amplification, two internal *Bsa*I sites in the DHFR UTRs of the *hxgprt* cassette were removed using the Q5 Site-Directed Mutagenesis Kit with primers P23/P24 and P25/P26. The resulting DNA parts were cloned into an ampR-p15A backbone in a four-part BASIC reaction, forming pCORe. All BASIC linkers used in the assemblies were synthesized by Biolegio and are listed in Supplementary Table 13.

### Transfections

All transfections were performed by electroporation using an Amaxa 4D-Nucleofector (Lonza) with programme ‘F1-115’. Transfections were carried out using freshly collected extracellular tachyzoites in P3 buffer (5 mM KCl, 15 mM MgCl_2_, 120 mM Na_2_HPO_4_/NaH_2_PO_4_ pH 7.2 and 50 mM d-mannitol).

### Stable parasite line generation

To generate the iKO strain for *Tg*Hypo (here referred to as RH *Tg*Hypo^iKO^), 10 µg of *Sca*I-linearized pG140::*Tg*Hypo-3 × HA was co-transfected with 10 µg of pSAG1::Cas9-U6::sg*Tg*Hypo(×2) into 5 × 10^6^ RHdiCreΔ*ku80*Δ*hxgprt* parasites^55^. Transgenic parasites were selected with 25 µg µl^−1^ mycophenolic acid and 50 µg µl^−1^ xanthine 24 h post-transfection, and individual resistant clones were obtained by limiting dilution. Successful 5′ and 3′ integration of the DNA construct at the endogenous *Tg*Hypo locus was confirmed by PCR using primer P27/P28 and P29/P30, respectively. Disruption of the endogenous *Tg*Hypo locus was confirmed using primers P31/P32. Rapamycin-induced excision of the integrated *Tg*Hypo iKO construct was verified using primers P33/P34.

To generate parasite strains ecotopically overexpressing RPL4 and cysteine-231 mutants of (here referred to as RHΔ*ku80*^RPL4^ and RH Δ*ku80*^RPL4(C231X)^, respectively) at the *UPRT* locus, 20 µg of *Pci*I-linearized pUPRT::FLAG-RPL4 or pUPRT::FLAG-RPL4^C231A/S/D/Y^ was co-transfected with 10 µg pSAG1::Cas9-U6::sgUPRT into 5 × 10^6^ RHΔ*ku80*Δ*hxgprt* parasites^56^. Transgenic parasites were selected with 5 µM 5-fluorodeoxyuridine, and individual resistant clones were obtained by limiting dilution. Integration at the 5′ and 3′ sites was validated using primers P35/P36 and P37/P38, respectively. Disruption of the endogenous UPRT was confirmed using primers P39/P40.

### iKO of TgHypo

Confluent HFF monolayers in T25 flasks were infected with ~2–5 × 10^6^ parasites for 4 h before treatment with 50 nM rapamycin or an equivalent volume of vehicle (DMSO) for 4 h. After washout, parasites were grown for at least 24 h before PCR or western blot analysis.

### SDS–PAGE and western blot analysis

Extracellular parasites were lysed RIPA buffer (150 mM NaCl, 50 mM Tris–HCl (pH 8.0), 1% Triton X-100, 0.5% sodium deoxycholate, 0.1% SDS and 1 mM EDTA) or mild lysis buffer (1% Triton X-100 and 0.1% SDS) supplemented with cOmplete Protease Inhibitor Cocktail (Roche) for 1 h on ice. Lysates were then centrifuged (21,000*g*, 30 min at 4 °C), and protein concentration in the supernatant was quantified using the Pierce BCA Protein Assay Kit (Thermo Scientific). Laemmli buffer was added to the lysate to 1× concentration (2% SDS, 10% glycerol, 5% 2-mercaptoethanol, 0.002% bromophenol blue and 125 mM Tris–HCl, pH 6.8) and boiled (95 °C, 5 min) before separation by SDS–PAGE on 12% polyacrylamide gels. Thirty micrograms of protein were typically loaded per lane. Proteins were transferred (20 V, 1 min; 23 V, 4 min; 25 V; 2 min) to nitrocellulose membranes using an iBlot 2 Dry Blotting System (Invitrogen). Membranes were briefly washed in PBS-T (0.1% Tween-20/PBS), blocked (5% skimmed milk/PBS-T, 1 h) and incubated with primary antibodies (1% BSA/PBS-T, overnight at 4 °C) at the following dilutions: mouse α-SAG1 (1:1,000, Invitrogen, D61S), rat α-HA (1:2,000, Roche, 3F10), rabbit α-*T. gondii* (1:2,000, Invitrogen, PA1-7252) and rabbit α-FLAG (1:2,000, Sigma, F7425). Following washing (PBS-T, 3×), membranes were incubated with HRP-conjugated secondary antibodies (1:5,000-10,000, Invitrogen) in 1% BSA/PBS-T for 1 h at room temperature. Protein bands were developed using the ECL Western Blotting Detection Reagent (GE Healthcare), and chemiluminescence was visualized using a ChemiDoc MP Imaging System (Bio-Rad). Relative band intensity was routinely quantified using ImageJ software. For analysis of RPL4 expression in RHΔ*ku80*^RPL4^ and RH Δ*ku80*^RPL4(C231X)^ parasites, the intensity of the ~55 kDa α-FLAG band (that is, RPL4) was normalized to the ~50 kDa band in the α-*T. gondii* loading control. The mean (± standard deviation (s.d.) relative band intensity of RPL4 cysteine mutants was then statistically compared with the RHΔ*ku80*^RPL4^ sample by one-way analysis of variance (ANOVA).

### Immunofluorescence microscopy

Confluent HFF monolayers grown on glass coverslips were seeded with ~100,000 parasites. Approximately 24 h post-infection, cells were fixed (4% paraformaldehyde for 15 min at room temperature) permeabilized (0.1% Triton X-100/PBS for 5–10 min) and blocked (3% BSA/PBS for 1 h at room temperature). Staining was performed for 1 h with primary antibodies at the following dilutions: mouse α-SAG1 (1:1,000, Invitrogen, D61S), rabbit α-HA (1:1,000, Cell Signaling Technology, C29F4), rabbit α-*T. gondii* (1:1,000, Invitrogen, PA1-7252) and mouse α-FLAG M2 (1:1,000, Sigma, F1804). Labelled proteins were stained for 1 h at room temperature using Alexa Fluor 488/594-conjugated goat antibodies (1:2,000, Invitrogen). Nuclei were stained using the intercalating DNA dye DAPI at 5 µg ml^−1^. Stained coverslips were mounted onto glass slides using VECTASHIELD Antifade Mounting Media (Vector Labs) and imaged on a Nikon Ti-E inverted microscope using NIS-Elements software. Images were acquired using an ORCA-Flash 4.0 camera and processed using ImageJ software.

### *T. gondii* plaque formation assay

Confluent HFF monolayers grown in six-well plates were seeded with 200–400 parasites, and plaques were left to form undisturbed for 6–7 days. For plaque assays involving conditional KO of *Tg*Hypo, parasites were first allowed to invade overnight before treating with 50 nM rapamycin or DMSO for 4 h and replacing the drug medium with standard culture medium. Monolayers were then fixed with ice-cold methanol for 10 min and stained with crystal violet stain (2.3% crystal violet, 0.1% ammonium oxalate and 20% ethanol) for 2 h. Plaques were enumerated manually, and statistical significance in plaque counts between rapamycin and DMSO-treated samples was tested using two-tailed unpaired Student’s *t*-tests with unequal variance. The data are presented as mean (± s.d.) counts.

### *P. falciparum* growth inhibition assay

Highly synchronized *P. falciparum* 3D7 cultures were diluted to 2% parasitaemia and 1% haematocrit before culturing in 96-well plates containing the appropriate concentration of drug. Wells containing DMSO and 10 µM cycloheximide were used as a negative and positive controls, respectively. Parasites were maintained at 37 °C under a gas mixture of 96% N_2_, 3% O_2_ and 1% CO_2_ for 72 h before freezing plates overnight at −80 °C. Thawed parasites were lysed in SYBR Green lysis buffer (20 mM Tris, 5 mM EDTA, 0.008% (w/v) saponin and 0.08% (v/v) Triton X-100, pH 7.5, 1:10,000 SYBR Green; Invitrogen) for 1 h at room temperature. Fluorescence was then measured using a Tecan M200 Infinite Pro microplate reader at 490 nm excitation and 520 nm emission. The percentage inhibition of asexual parasite growth was calculated relative to control treatments.

### Design and optimization of the CORe platform

**Extended Data Fig. 9:**
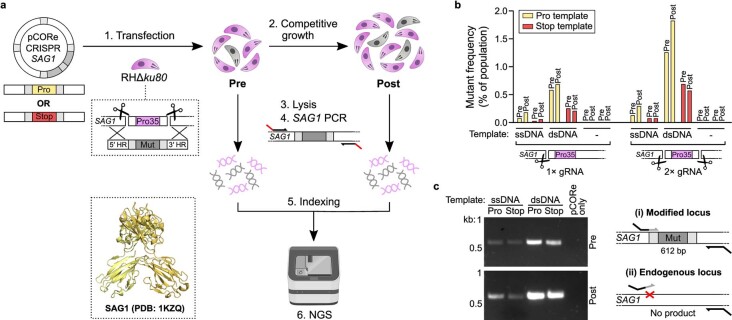
Optimal editing efficiency at *SAG1* is achieved using a dual gRNA strategy with double-stranded template conformation. **a**, Workflow used for assessing the integration efficiency of templates for site-directed mutagenesis of *SAG1* (P35) with a synonymous recodonized proline (wildtype) or stop codon (knockout). **b**, Frequency of *SAG1*-modified parasites before (‘Pre’) and after (‘Post’) a period of competitive lytic growth. Templates were provided in either single (ssDNA) or double-stranded (dsDNA) conformation and transfected with single or dual gRNA-containing pCORe CRISPR plasmids. For each mutation type, a maximum integration frequency (1–2%) is achieved following transfection of dual gRNA plasmids with dsDNA templates. **c**. Template-specific PCR from dual gRNA samples showing selective amplification of proline mutant DNA. Data represents a single experiment (n = 1).

The design of the CORe workflow begins with the identification and selection of paired CRISPR guide RNA (gRNA) sequences that target the Cas9 nuclease to sites 5′ and 3′ of a target cysteine codon. As demonstrated in *Caenorhabditis elegans*^57^, we reasoned that a dual gRNA strategy would provide positive selection towards HDR-mediated integration of mutational templates for our essential gene subset, as the lack of repair of two double-strand breaks (DSBs) in an essential gene should be refractory to growth. To test this hypothesis, the frequency of mutants following mutagenesis of an N-terminal proline codon in surface antigen gene1 (*SAG1*) was compared using single or dual gRNAs in combination with single- or double-stranded strand donor repair templates (Extended Data Fig. 9a). These experiments revealed that dual gRNAs in combination with double-stranded templates provided the highest integration efficiency in the absence of any selectable marker. As anticipated, in the absence of drug selection, the frequency of mutants was low (Extended Data Fig. 9b). The potential negative impact of this upon quantitation of integration events was circumvented through the inclusion of recodonized sequence within the donor template. This allowed for integration-selective priming and therefore generation of PCR amplicons of modified genomic loci for downstream NGS analyses (Fig. 2a and Extended Data Fig. 9c). The protein-centric CRISPR guide design tool, CRISPR-TAPE^58^, was used to simplify and accelerate the gRNA identification and selection process for target cysteines. Accommodating the need for high-throughput multiplexed vector construction, BASIC^53^ was adapted to our sequences and used for facile, modular and scalable production of all transfection vectors, with dual gRNA cassettes and Cas9 encoded on the same vector as previously reported (Extended Data Fig. 3a)^25,59^. The RH∆*ku80∆hxgprt* NHEJ-deficient parasite strain was used to further promote HDR^56^.

Donor repair templates were designed to (1) destroy the protospacer adjacent motif (PAM) and/or gRNA seed sequence required for Cas9 targeting and so prevent further modification of the site following integration; (2) provide a recodonized stretch of sequence proximal to the target cysteine for the generation of integration-specific amplicons at mutated sites. Transfection with the dual gRNA vector introduces DSBs 5′ and 3′ of the target cysteine. The excised locus is subsequently repaired using one of the donor templates, producing a mixed mutant pool, which is sampled shortly after transfection for subsequent genomic DNA extraction (‘Pre’ sample) (Fig. 2a). For each reactive cysteine candidate, *T. gondii* tachyzoites are co-transfected with a single cysteine-targeting dual gRNA plasmid and all five donor templates for HDR (Fig. 2a). The repair templates encoded for either a WT synonymous replacement of the target cysteine, a stop codon or one of the three amino-acid substitution options.

Following transfection, the mixed population of mutants grow competitively, and are sampled for genomic extraction (‘Post’ sample) (Fig. 2a). Where the DSB is repaired using the synonymous WT template, parasites are expected to grow normally. In instances where the stop codon template is integrated, the gene coding sequence is disrupted, with parasite growth anticipated to be attenuated equivalent to a KO^26^. After quantitative deep sequencing of integration-specific amplicons encompassing a target cysteine, the frequency of reads for a given mutant in the Post sample (*f*_Post_) is normalized to Pre (*f*_Pre_) to derive fitness scores (Fs) that reflect the viability of parasites during competitive lytic growth. The Fs for the amino-acid mutants are benchmarked against the synonymous WT and stop codon mutants. This provides a quantitative assessment of the contribution of an individual cysteine to protein function in live cells, using mutant cell fitness as a measurable phenotype and NGS reads as the readout. Multiplexing of CRISPR vector construction with BASIC, 96-well-plate-based transfections and automated NGS sample preparation workflow enables hundreds of targets to be functionally interrogated in parallel.

### CORe plasmid and template library design and construction

gRNAs were searched against the *T. gondii* GT1 genome (release 46; www.toxodb.org) using the ‘position-specific’ function of CRISPR-TAPE (version 1.0.0) (ref. ^58^). Briefly, gRNAs binding in close proximity to a target cysteine codon were identified by applying a search distance threshold of ±200 nt. For each codon, two gRNAs binding at sites 5′ and 3′ of the residue were then selected. Selection criteria was based on the number of potential off-target sequences, %GC content and the ability to introduce synonymous PAM or guide blocking mutations at the target genomic sequence. gRNAs were synthesized by Twist as a fragment containing a U6 promoter and flanking BASIC Prefix and Suffix sequences, and independently cloned into *Bsa*I sites of a kan^R^-pMB1 storage plasmid, pTwist Kan (High Copy). For each target cysteine, the corresponding 5′- and/or 3′-binding gRNA fragment were subcloned into pCORe in a three-part BASIC reaction, replacing the mScarlett counterselection cassette and generating the pCORe-CRISPR plasmid. The sequences of all gRNA fragments are listed in Supplementary Table 13.

Donor templates for mutation of target cysteines were synthesized as 300 bp double-stranded fragments by Twist. Lyophilized templates were re-hydrated and used directly in the transfection. For the *SAG1* experiments, 70 bp single-stranded oligonucleotides were used and hybridized to generate double-stranded templates. For each cysteine codon, five templates were designed to incorporate single unique mutations: a recodonized cysteine codon, alanine, serine, tyrosine or a stop codon. Mutation sites were flanked by regions of synonymous recodonized sequence to (1) enable specific detection of cysteine mutants by PCR, and (2) introduce blocking mutations at the PAM and/or gRNA seed sequence to prevent re-excision of modified genomic loci. The recodonized regions were designed to incorporate the most frequently used codons possible (according to the codon usage bias for *T. gondii* listed on the IDT Codon Optimization Tool; www.eu.idtdna.com/CodonOpt), while maintaining a minimum of 30% unique bases in the PCR priming sequence. Recodonization was avoided or minimized at intron–exon junctions to avoid interference with messenger RNA splicing. Homology regions were incorporated on either end of templates to promote genomic integration of mutational templates by HDR. The sequences of all mutational templates are listed in Supplementary Table 13.

### CORe mutagenesis screens

Transfections were carried out in 16-well Nucleocuvette strips using the Amaxa 4D-Nucleofector X-Unit (Lonza). For the primary optimized CORe screen, 7 µg of pCORe-CRISPR and 0.2 µg of each of the five corresponding mutational templates (equivalent to a ~1:5 plasmid-to-template molar ratio) were co-transfected into 1 × 10^6^ RHΔ*ku80*Δ*hxgprt* parasites^56^. For the saturation mutagenesis experiments, 7 µg of pCORe-CRISPR and 0.5 µg of each of the 21 mutational templates (~1:5 plasmid-to-template molar ratio) were transfected. For the *SAG1* experiments, 6 µg of pCORe-CRISPR and 2 µg of a single template were transfected (~1:100 plasmid-to-template molar ratio). Transfected parasites were expanded in HFF monolayers grown in 24-well plates and allowed to egress naturally 3 days after infection. Approximately 2 × 10^6^ of the egressed parasites were used to infect confluent HFF monolayers in six-well plates, and the remaining parasites (~2 × 10^6^) were pelleted and frozen for genomic DNA extraction as the initial ‘Pre’ mutant population. Parasites were allowed to egress naturally 5 days after infection and similarly collected as the ‘Post’ mutant population. Parasite genomic DNA from frozen cell pellets was extracted using the DNeasy Blood & Tissue Kit (Qiagen) for downstream NGS library preparation.

### Illumina library preparation, sequencing and data analysis

Genomic DNA libraries were prepared similarly to the 16S Metagenomic Sequencing Library Preparation guide (Illumina). Briefly, for each target cysteine, a ~600–800 bp fragment targeting the modified genomic locus was PCR amplified from parasite DNA. For the *SAG1* experiments, the amplicons were designed to encompass the template integration site of both modified and unmodified loci. All primers were designed to include overhanging Illumina adapter sequences and are listed in Supplementary Table 12 (P41–P190). The resulting amplicon was purified using AMPure XP magnetic beads (Beckman Coulter). Dual indices and sequencing adapters were then ligated to the purified products using the Nextera XT Index Kit (Illumina). Indexed amplicons were then purified using AMPure XP beads, and quantified using the Qubit dsDNA HS/BR Assay Kits (Invitrogen), or the QuantiFluor ONE dsDNA System (Promega). Indexed amplicons were pooled at equimolar concentration, and the size and purity of the resulting library was assessed on a TapeStation 2200 with the D1000 ScreenTape System (Agilent). The transfer of reagents used for the purification and indexing of amplicons was performed using acoustic liquid handling (Echo 525, Labcyte). Pooled libraries were sequenced using an Illumina NextSeq 500 75PE Mid Output run with a PhiX spike-in of 10%. For the initial screen, indexed amplicons encompassing all 74 electrophile-sensitive cysteines encoding five substitutions/cysteine were indexed for multiplexing onto a single run, that is, 370 unique substitutions were analysed on a single NGS run. Independent biological replicates of the screen were analysed on independent NGS runs. For the saturation substitution analysis of the 10 translation-associated cysteines, all 210 substitutions (10 cysteines with 21 substitutions/cysteine) were indexed for multiplexing, and all three biological replicates analysed on a single run, that is, 630 substitutions were analysed on a single NGS run. Following acquisition, sequencing data were demultiplexed using CASAVA 2.17 and analysed using the Galaxy web server (www.usegalaxy.org). For each uniquely indexed sample, the sequences were concatenated and separated by each template variant to determine the read counts of the different mutation types. Fs values, representing the change in frequency of each mutant variant, were then calculated by dividing the percent proportion of reads in the Post population sample to the Pre. The differences in Fs values of the non-synonymous mutations were normalized and statistically tested against the recodonized cysteine mutation by one-way ANOVA. Deleterious and gain-of-function mutations were classified by statistically significant normalized Fs of <0.66 and >1.33, respectively. Fitness-conferring cysteines were defined as sites containing at least one deleterious or gain-of-function substitution type.

### Cysteine labelling and click chemistry

Cell pellets of *T. gondii* RHΔ*ku80*Δ*hxgprt* parasites were lysed by sonication in PBS (pH 7.4) and soluble fractions separated by centrifugation at 3,500*g* for 5 min. Protein concentrations were determined using the DC Protein Assay Kit (Bio-Rad) and a NanoDrop 2000c Spectrophotometer (Thermo Scientific). Proteome samples diluted to 2 mg ml^−1^ were treated with 10 or 100 µM IA-alkyne (from 1 mM and 10 mM stocks in DMSO, respectively) and incubated for 1 h at room temperature with rotation. The labelled proteins were then subject to click chemistry by addition of 100 µM Azo-L or Azo-H, 1 mM TCEP, 100 µM TBTA and 1 mM CuSO_4_ (final concentrations). Click reactions were incubated for 1 h at room temperature with shaking. The Azo-L/H-labelled protein samples were then precipitated by adding trichloroacetic acid to 10% (v/v) concentration. After overnight storage at −80 °C, precipitated proteins were pelleted by centrifugation (17,000*g*, 10 min), washed three times with chilled MeOH and resolubilized in 1.2% SDS in PBS by gentle sonication and heating (80 °C, 10 min).

### Enrichment and on-bead digestion

Labelled proteome samples were diluted to 0.2% SDS with PBS. The resulting samples were then added to 100 µl of Pierce streptavidin beaded agarose resin (Thermo Scientific) and incubated overnight at 4 °C followed by a further 2 h at room temperature. Protein-bound beads were washed with 1× 0.2% SDS in PBS, 3× PBS and 3× H_2_O before resuspending in 6 M urea in PBS + 10 mM DTT and incubating at 65 °C for 15 min. Reduced samples were then alkylated by adding IAA to a final concentration of 20 mM and incubating for 30 min at 37 °C with rotation. Samples were diluted three-fold with PBS and centrifuged (1,400*g*, 2 min) to pellet the beads. The beads were resuspended in a mixture of 200 µl of 2 M urea in PBS, 1 mM CaCl_2_ and 2 µg trypsin (Promega) and incubated overnight at 37 °C. The beads were separated from the digest by centrifugation and washed three times with PBS and three times with H_2_O. Azo-labelled peptides were then cleaved by adding 50 mM sodium hydrosulfite (Na_2_S_2_O_4_) and rotating at room temperature for 1 h. Eluted peptides were then collected from the supernatant, and Na_2_S_2_O_4_ cleavage was repeated twice more to fractionate the sample. Between each cleavage, the beads were washed with 2× H_2_O and combined with the previous elution. Formic acid was added to the sample to 20% (v/v) concentration before storing at −20 °C until MS analysis.

### LC–MS/MS analysis, peptide identification and quantification

Liquid chromatography with tandem mass spectrometry (LC–MS/MS) analysis was performed on an LTQ-Orbitrap Discovery mass spectrometer (Thermo Scientific) coupled to an Agilent 1200 Series HPLC. Azo digests were pressure loaded onto 250 µm fused silica desalting columns (Agilent) packed with 4 cm Aqua C18 reverse phase resin (Phenomenex). Peptides were then eluted onto a biphasic column consisting of 100 µm fused silica packed with 10 cm C18 and 4 cm PartiSphere SCX resin (Whatman) following a five-step multi-dimensional LC/LC–MS/MS protocol (MudPIT). Each step used a salt push (0%, 50%, 80%, 100% and 100%) followed by an elution gradient of 5–100% Buffer B in Buffer A (Buffer A: 95% H_2_O, 5% MeCN and 0.1% formic acid; Buffer B: 20% H_2_O, 80% MeCN and 0.1% formic acid) at a flow rate of 250 nl min^−1^. Eluted peptides were injected into the mass spectrometer by electrospray ionization (spray voltage set at 2.75 kV). For every MS1 survey scan (400–1800 *m*/*z*), eight data-dependent scans were run for the *n*th most intense ions with dynamic exclusion enabled.

The generated tandem MS data were searched using the SEQUEST algorithm^60^ against the *T. gondii* database (GT1 proteome), *Toxo*DB (www.toxodb.org). A static modification of +57.02146 on cysteine was specified to account for alkylation with IAA. Variable modifications of +456.2849 and +462.2987 were further assigned on cysteine to account for the probe modification with the isotopically light (Azo-L) and heavy (Azo-H) variant of the IA-alkyne-Azo adduct, respectively. Output files from SEQUEST were filtered using DTASelect 2.0. Quantification of isotopic light:heavy ratios was performed using the CIMAGE quantification package as previously described^4^. Overlapping tryptic peptides containing the same labelled cysteine (but different charge states or tryptic termini) were grouped and the median reported as the final light:heavy ratio (*R*). *R* values were averaged across biological replicates, and peptides with relative s.d. of the ≥ 50% *R* value were removed.

### Bioinformatics analysis of electrophile-sensitive cysteine dataset

Functional annotation of electrophile-sensitive cysteine-containing proteins was carried out using BLASTP, Gene Ontology and InterPro searches within Blast2GO 5 PRO software^61^. Consensus protein sequences were BLASTP searched against the non-redundant (nr) NCBI protein database using an *E*-value cut-off of 10^−6^. Gene Ontology terms (molecular function, biological process and subcellular localization) were then mapped from the top 20 hits and merged with annotations derived from the InterPro database (www.ebi.ac.uk/interpro). Assignments were further optimized using Annex augmentation. Enrichment of annotations was assessed using Fisher’s exact test against the *T. gondii* proteome (strain GT1; UniProt Taxonomy ID 507601) at <0.05 false discovery rate. All hypothetical proteins with no functional annotations were removed from data before performing enrichment analyses.

Assessments of gene essentiality in targets containing electrophile-sensitive cysteines were carried out using published phenotype scores^25^. The distribution of phenotype scores of gene products between datasets was statistically compared by two-tailed *t*-tests. To ensure comparison of only relevant tachyzoite genes, all genes upregulated in bradyzoite stage (displaying ≥5-fold higher expression in chronic versus acute infection in vivo)^62^ were removed before analysis.

For conservation analyses of electrophile-sensitive cysteines, orthologues of the associated protein were identified from orthologue groups classified on OrthoMCL^63^. Conservation of a given residue was assessed following BLASTP alignment of the orthologous protein sequence against the *T. gondii* template sequence. Scores were assigned to each alignment on the basis of the presence or absence of a matched cysteine; a score of 3 was assigned to conserved cysteines, 1 for no conservation and 0 if no protein was identified in the orthologue group for a given species. Conservation scores were determined for each cysteine by summing of the scores across the analysed species.

### Design and physico-chemical assessment of the acrylamide fragment library

One-hundred compounds were initially filtered from a large collection of >1,000 cysteine-reactive fragments using the FragFp diversity algorithm in DataWarrior (version 5.5.0, OpenMolecules). This subset was further filtered to 88 acrylamide electrophiles on the basis of the following physico-chemical properties, derived from their SMILES strings using Molecular Operating Environment (Chemical Computing Group):

- Molecular weight (gmol^−1^): 150–320
- Number of aromatic rings: ≤2
- Hydrogen bond acceptors: 2–5
- Hydrogen bond donors: ≤2
- clog*P*: −2 to 3

The principal moment of inertia (PMI) was calculated for each fragment to assess the molecular shape diversity of the library. The SMILES strings of the library were imported into Molecular Operating Environment, and a conformational search was performed using the following parameters: force field, MMFF94x; method, stochastic; rejection limit, 200; iteration limit, 10,000; RMS gradient, 0.005; MM iteration limit, 500; allow amide bond rotation, yes; allow unconstrained double bond rotation, yes; enforce chair conformations, yes; refine with QM, no; RMSD limit, 0.15; energy window, 7; conformation limit, 1. normalized PMI values (npr1, *I*_1_/*I*_3_; npr2, *I*_2_/*I*_3_) were visualized in triangular graphs, where *x* (*I*_1_/*I*_3_) and *y* (*I*_2_/*I*_3_) values of 0,1, 0.5,0.5 and 1,1 represent a perfect rod, disc and sphere, respectively.

### IVT assay

Pellets of *P. falciparum* 3D7 or HEK 293F cells were suspended in 1× pellet volume of lysis buffer supplemented with 20 U of human placental RNase inhibitor and cOmplete EDTA-free Protease Inhibitor Cocktail (Roche). Resuspended cells were then transferred to a pre-chilled nitrogen cavitation chamber (Parr Instrument Company) and incubated on ice at 1,500 PSI for 60 min. Following release from the chamber, the crude lysate was clarified by differential centrifugation (15 min at 10,000*g* and 4 °C, followed by 15 min at 30,000*g* and 4 °C). Protein concentration was determined using a NanoDrop (Thermo Scientific) at 280 nm and adjusted to 12 mg ml^−1^ before storage at −80 °C.

Before performing IVT assays, low-bind 384-well plates (Corning) were printed (D300e Digital Dispenser, Tecan) with compounds dissolved in DMSO to be assayed at 0.5% of the total assay volume. Five microlitres of clarified *P. falciparum* or HEK 293F lysate was then added to each well, followed by 4.5 μl l-amino acids (each at 200 μM in 45 mM HEPES pH 7.45, 100 mM potassium acetate, 1.5 mM magnesium acetate, 1 mM DTT, 20 U human placental RNase inhibitor, 15 μM leupeptin, 1.5 mM ATP, 0.15 mM GTP, 40 U ml^−1^ creatine phosphokinase and 4 mM creatine phosphate (Thermo Scientific), 2% (w/w) PEG3000, 1 mM spermidine and 0.5 mM folinic acid) and 0.45 μl of purified red click-beetle luciferase (CBG99) mRNA (1 μg μl^−1^). CBG99 mRNA was transcribed from expression plasmids pH-CBG99-H (for use in *P. falciparum* assays) or pT7CFECBG99 (HEK 293F assays) as previously described^41^. Prepared plates were incubated at 32 °C for 1 h 40 min before adding 10 µl of 45 mM HEPES pH 7.45, 1 mM magnesium chloride, 1 mM ATP, 5 mM DTT, 1% (v/v) Triton-X, 10 mg ml^−1^ BSA, 1× Reaction Enhancer (Thermo Scientific), 1 mg ml^−1^ d-luciferin (Thermo Scientific) and 0.5 mM cycloheximide. Luminescence was measured across each well using a Tecan M200 Infinite Pro microplate reader heated to 37 °C. Each fragment screen was run as two independent assays with DMSO and cycloheximide (10 µM) used as negative and positive controls, respectively. The percent of translation inhibition (%*I*) for each test compound was calculated from raw luminescence values as in equation (1) (where *V* is the measured value, *µ* is the mean value, and d, p and n represent the test compound, positive control and negative control, respectively).

1 $$\% {I} = 100 - \left( {\frac{{\left( {{V}_{{{\mathrm{d}}}} - {\mu}_{{{\mathrm{p}}}}} \right)}}{{\left( {{\mu }}_{{{\mathrm{n}}}} - {\mu}_{{{\mathrm{p}}}} \right)}} \times 100} \right)$$

The robustness of plate-based screens were determined by *Z*-factors (*Z*′) from the positive and negative controls as in equation (2) (where *σ* is the s.d., and 0.5 ≤ *Z*′ reflects acceptable assay quality).

2 $${Z}\prime = 1 - \left( {3 \times \frac{{\left( {{\sigma }}_{{{\mathrm{p}}}} + {\sigma}_{{{\mathrm{n}}}} \right)}}{{\left( {{\mu }}_{{{\mathrm{p}}}} - {\mu}_{{{\mathrm{n}}}} \right)}}} \right)$$

### Protein structures, homology modelling and folding analyses

Solved protein structures were downloaded from the RCSB Protein Data Bank (www.rcsb.org). Homology models were predicted from primary protein sequences using AlphaFold 2.0 (ref. ^35^) or the Phyre2 web portal^36^; only models constructed with 100% confidence and ≥40% sequence identity (Phyre^2^) or ≥70 pLDDT (AlphaFold) across ≥70% of the sequence were used for downstream analyses. Structural analyses of the impact of cysteine substitutions on protein folding stability in silico were predicted using FoldX 5.0 (http://foldxsuite.crg.eu)^37^. For each cysteine mutant, differences in the free energy of folding (ΔΔ*G*) relative to the WT structure was determined using the ‘PositionScan’ command as in equation (3) (where Δ*G*_mutant_ and Δ*G*_WT_ reflect the folding free energy of the mutant and WT protein models, respectively).

3 $${\Delta}{\Delta}G = {\Delta}G_{{{\mathrm{mutant}}}} - {\Delta}G_{{\mathrm{WT}}}$$

### Statistical analysis

Statistical tests were performed using GraphPad Prism 8.0 as described in the individual experimental sections above. *P* value significance thresholds were set at: ****P < 0.0001, ***P < 0.001, **P < 0.01 and *P < 0.05. All statistically significant results are annotated with a line and asterisk(s) in the graphs.

### General software

Schematics were created using Adobe Illustrator (version 22.1) and Inkscape (version 0.92.3). Chemical structures were drawn in ChemDraw Professional (version 18.0). PyMOL (version 2.1.1) was used to generate images of 3D protein structures.

### Reporting summary

Further information on research design is available in the Nature Research Reporting Summary linked to this article.

## Supplementary information

Supplementary Information Supplementary Figs. 1–4, discussion and references.

Reporting Summary

Supplementary Table Supplementary Table 1. isoTOP-ABPP MS data. Supplementary Table 2. List of electrophile-sensitive cysteines in *T. gondii*. Supplementary Table 3. List of highly electrophile-sensitive cysteines in *T. gondii*. Supplementary Table 4. Functional enrichment analysis of highly electrophile-sensitive cysteine-containing genes in *T. gondii*. Supplementary Table 5. Conservation analysis of highly electrophile-sensitive cysteines in *T. gondii*. Supplementary Table 6. Meta-analysis of cysteine mutation causing destabilization of PPIs in cancer-associated genes. Supplementary Table 7. CORe datasets for highly electrophile-sensitive cysteines in *T. gondii*. Supplementary Table 8. CORe datasets for saturation mutagenesis of highly electrophile-sensitive ribosomal cysteines in *T. gondii*. Supplementary Table 9. Acrylamide fragment library SMILES. Supplementary Table 10. Physico-chemical properties of the acrylamide fragment library. Supplementary Table 11. Fragment screen data against IVT assay of *P. falciparum* and HEK293 cell lysates. Supplementary Table 12. Primers used in the study. Supplementary Table 13. Synthetic DNA used in the study.

## Data Availability

As far as possible, all raw data supporting the findings in this study are available within the Article and its Supplementary Information files. Data obtained from sequencing have been deposited in the Sequence Read Archive (www.ncbi.nlm.nih.gov/sra) under accession number PRJNA860585. The MS proteomics data have been deposited to the ProteomeXchange Consortium via the PRIDE partner repository with the dataset identifier PXD035658. Existing data associated with the gene IDs in this project are available from ToxoDB (https://toxodb.org/toxo/app). Additional unprocessed data are available from the corresponding author upon request. Source data are provided with this paper.

## Source data

Source Data Fig. 1 Statistical data.
Source Data Fig. 2 Statistical data.
Source Data Fig. 2 Unprocessed agarose gels.
Source Data Fig. 3 Statistical data.
Source Data Fig. 4 Statistical data.
Source Data Extended Data Fig. 1 Statistical data.
Source Data Extended Data Fig. 2 Statistical data.
Source Data Extended Data Fig. 2 Unprocessed agarose gels and western blots.
Source Data Extended Data Fig. 4 Statistical data.
Source Data Extended Data Fig. 6 Statistical data.
Source Data Extended Data Fig. 7 Statistical data.
Source Data Extended Data Fig. 8 Statistical data.
Source Data Extended Data Fig. 8 Unprocessed agarose gels and western blots.
