# Supplementary material for: CRISPR-based oligo recombineering prioritizes apicomplexan cysteines for drug discovery
Source: Nat Microbiol. 2022 Oct 20;7(11):1891–905. doi: 10.1038/s41564-022-01249-y (PMC9613468; doi:10.1038/s41564-022-01249-y)
Supplement: Supplementary file 1 — Supplementary Figs. 1–4, discussion and references. [file 41564_2022_1249_MOESM1_ESM.pdf]

nature microbiology

Article https://doi.org/10.1038/s41564-022-01249-y

CRISPR-based oligo recombineering prioritizes apicomplexan cysteines for drug discovery

In the format provided by the authors and unedited

Supplementary information table of contents:

Supplementary Fig. 1: Comparison of traditional and CORe approaches for functional interrogation of amino acids through site-directed mutagenesis. 2

Supplementary Fig. 2: Integration-specific PCR enables selective amplification of CORe mutant DNA across diverse genomic loci. 3

Supplementary Fig. 3: Hyperreactive cysteines in *T. gondii* targeted by CORe exhibit reproducible and diverse mutational profiles. 7

Supplementary Fig. 4: Electrophile-sensitive cysteines of ribosome proteins in *T. gondii* display diverse mutational tolerance. 10

Supplementary discussion 12

Supplementary references 14

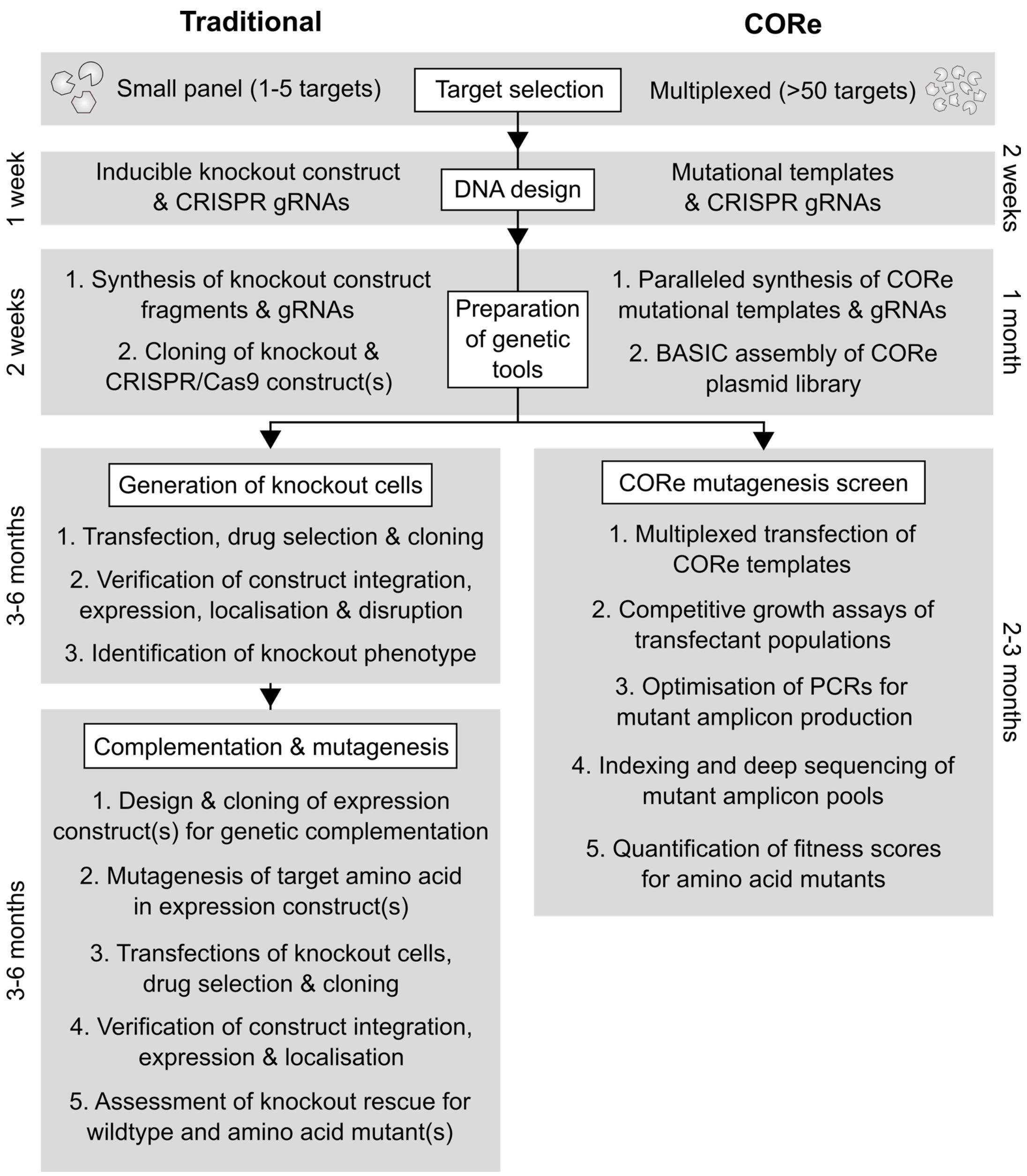

Supplementary Fig. 1: Comparison of traditional and CORe approaches for functional interrogation of amino acids through site-directed mutagenesis. Flowchart depicts the workflows for each approach, with the typical times for each experimental process indicated. It should be noted that the timescales presented for CORe are based on the results from this study, while timescales presented for the alternative approaches are only a best-estimate and will vary between individual researchers.

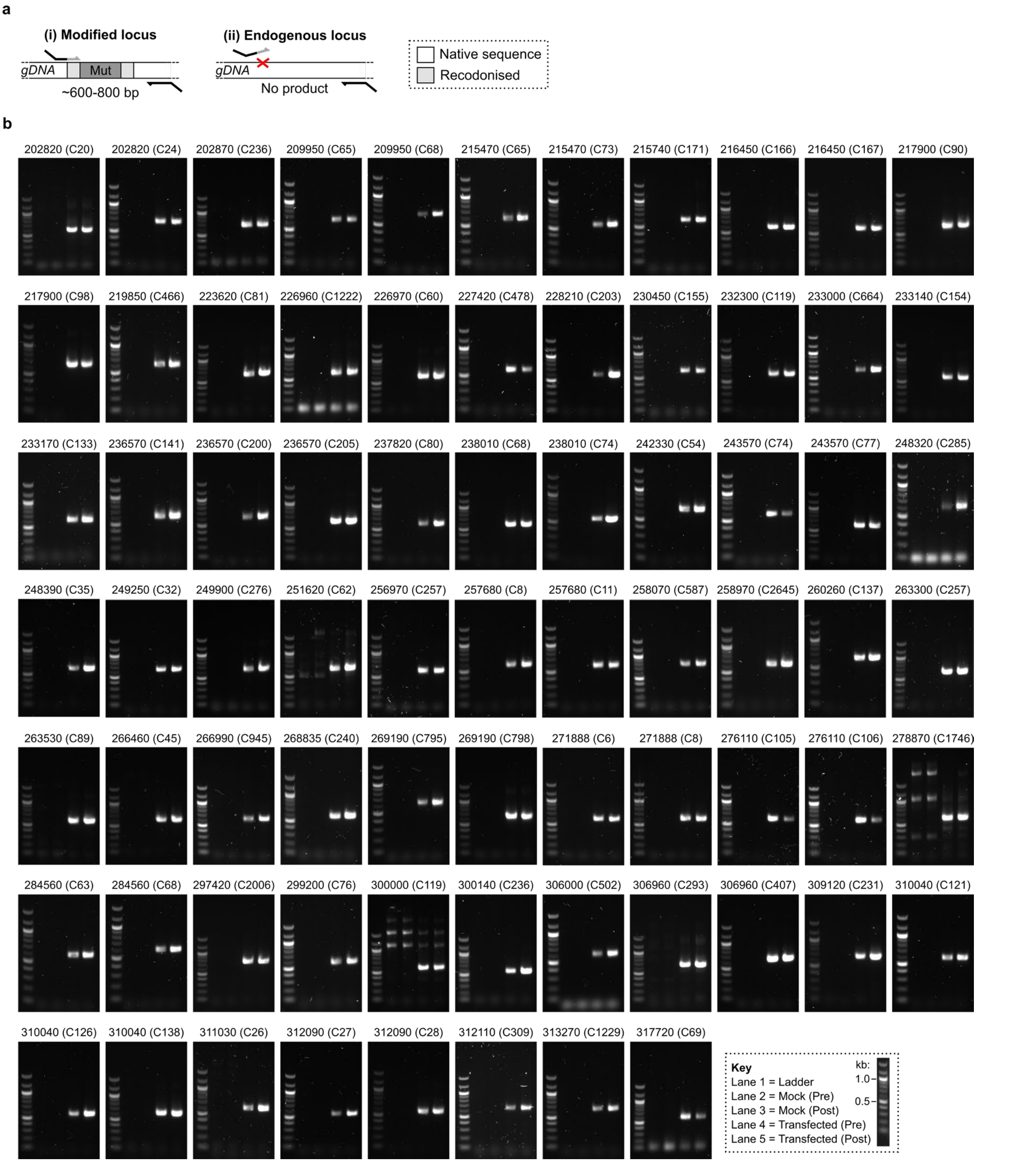

Supplementary Fig. 2: Integration-specific PCR enables selective amplification of CORe mutant DNA across diverse genomic loci. a, General PCR strategy for generating amplicons encompassing the modified cysteine loci of mutant parasites. Specific amplification of modified vs. endogenous genomic loci is achieved by priming regions of unique recodonized sequence in the integrated mutational templates. **b**, Integration-specific amplicons for 74 cysteines targeted for mutagenesis via CORe. For all targets, mutants are detected in both 'Pre' and 'Post' timepoints, and no product is generated in mock-transfected parasite populations. Gel scans for TGGT1_227420 (C478) and TGGT1_257680 (C8/C11)

represent those displayed in Figure 2c and are included for completion. Image scans shown are representative of results from three independent experiments.

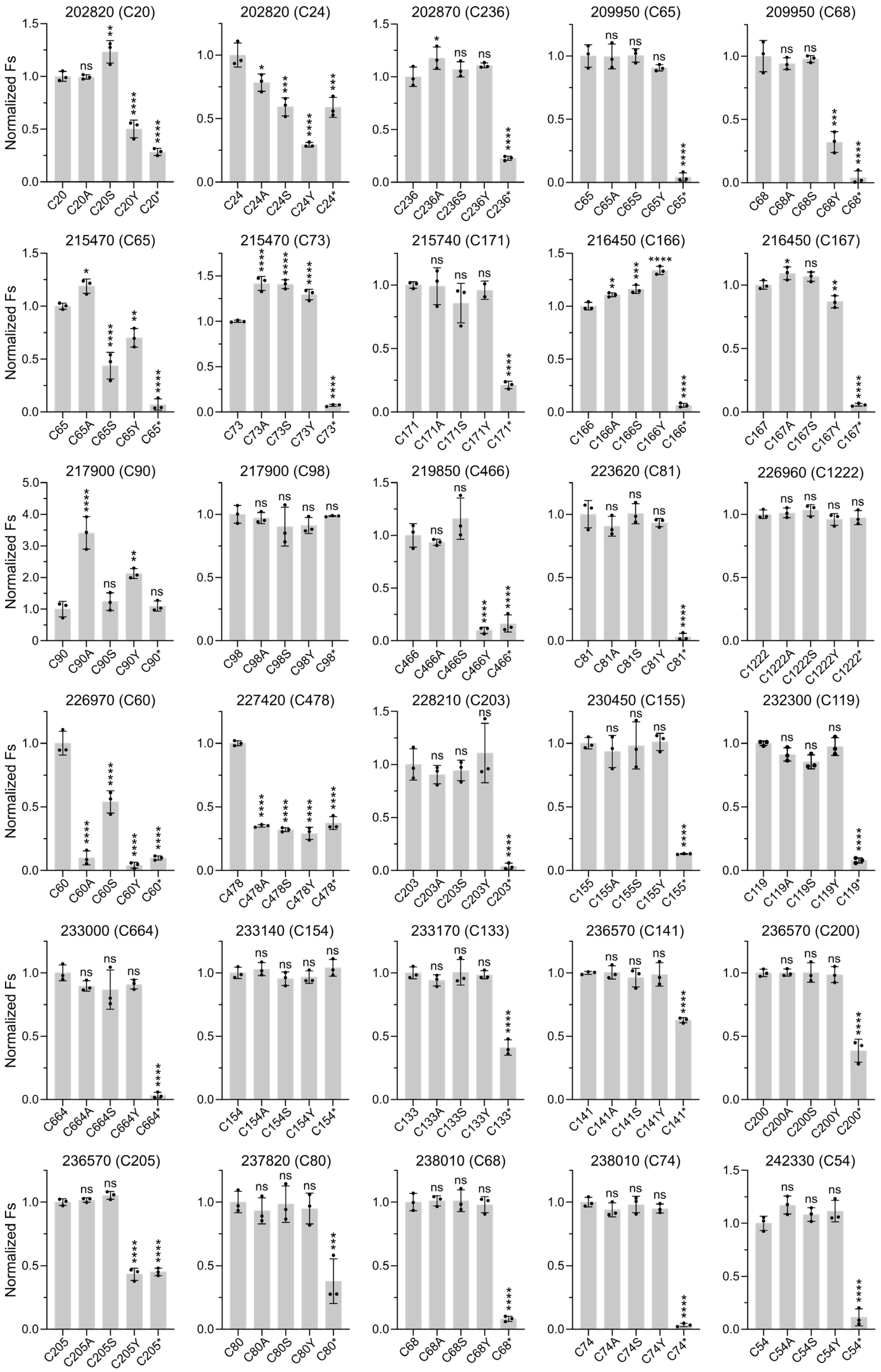
202820 (C20)
202820 (C24)
202870 (C236)
209950 (C65)
209950 (C68)
215470 (C65)
215470 (C73)
215740 (C171)
216450 (C166)
216450 (C167)
217900 (C90)
217900 (C98)
219850 (C466)
223620 (C81)
226960 (C1222)
226970 (C60)
227420 (C478)
228210 (C203)
230450 (C155)
232300 (C119)
233000 (C664)
233140 (C154)
233170 (C133)
236570 (C141)
236570 (C200)
236570 (C205)
237820 (C80)
238010 (C68)
238010 (C74)
242330 (C54)
Normalized Fs

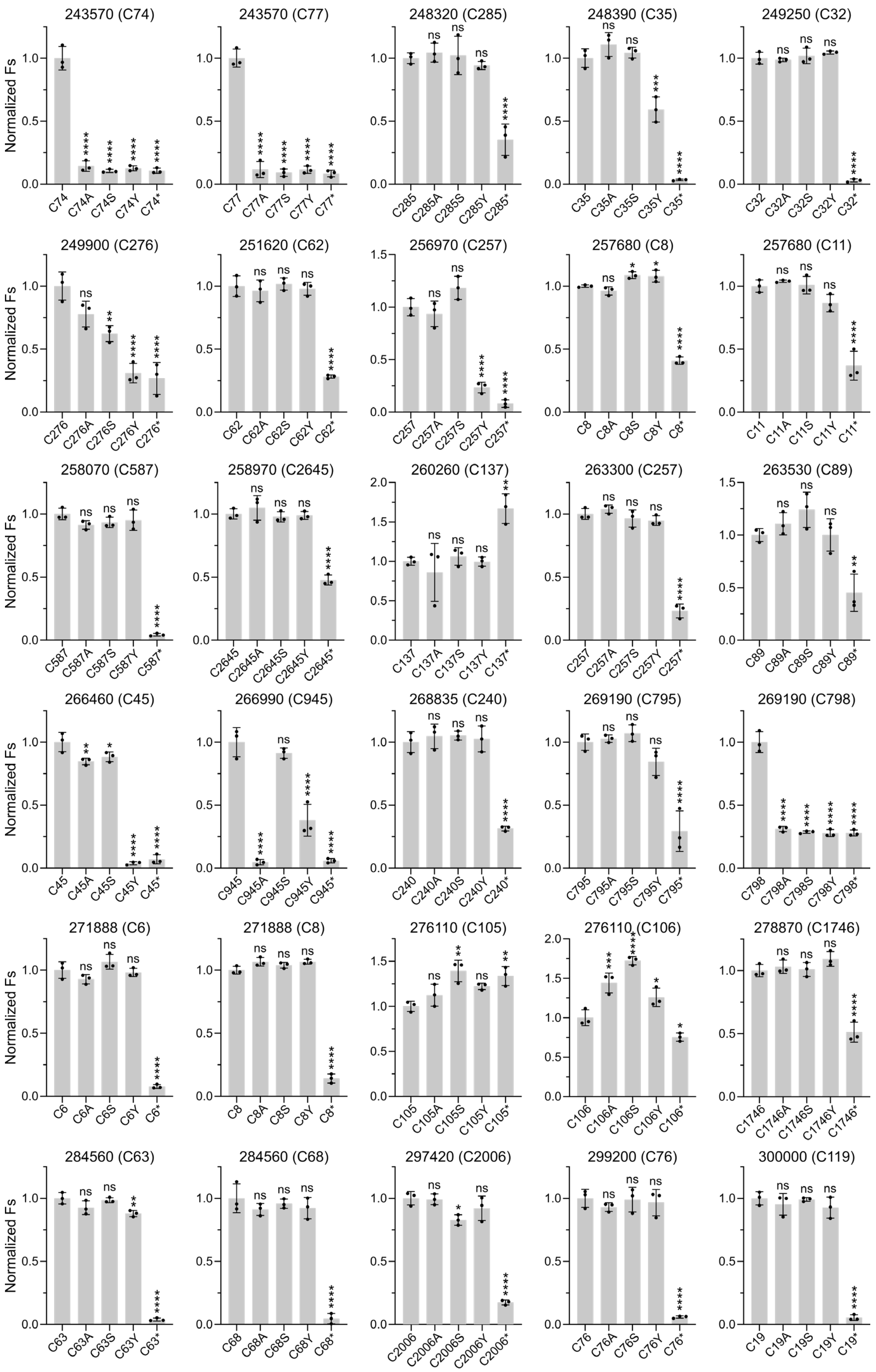
243570 (C74)
C74 C74A C74S C74Y C74*
243570 (C77)
C77 C77A C77S C77Y C77*
248320 (C285)
C285 C285A C285S C285Y C285*
248390 (C35)
C35 C35A C35S C35Y C35*
249250 (C32)
C32 C32A C32S C32Y C32*
249900 (C276)
C276 C276A C276S C276Y C276*
251620 (C62)
C62 C62A C62S C62Y C62*
256970 (C257)
C257 C257A C257S C257Y C257*
257680 (C8)
C8 C8A C8S C8Y C8*
257680 (C11)
C11 C11A C11S C11Y C11*
258070 (C587)
C587 C587A C587S C587Y C587*
258970 (C2645)
C2645 C2645A C2645S C2645Y C2645*
260260 (C137)
C137 C137A C137S C137Y C137*
263300 (C257)
C257 C257A C257S C257Y C257*
263530 (C89)
C89 C89A C89S C89Y C89*
266460 (C45)
C45 C45A C45S C45Y C45*
266990 (C945)
C945 C945A C945S C945Y C945*
268835 (C240)
C240 C240A C240S C240Y C240*
269190 (C795)
C795 C795A C795S C795Y C795*
269190 (C798)
C798 C798A C798S C798Y C798*
271888 (C6)
C6 C6A C6S C6Y C6*
271888 (C8)
C8 C8A C8S C8Y C8*
276110 (C105)
C105 C105A C105S C105Y C105*
276110 (C106)
C106 C106A C106S C106Y C106*
278870 (C1746)
C1746 C1746A C1746S C1746Y C1746*
284560 (C63)
C63 C63A C63S C63Y C63*
284560 (C68)
C68 C68A C68S C68Y C68*
297420 (C2006)
C2006 C2006A C2006S C2006Y C2006*
299200 (C76)
C76 C76A C76S C76Y C76*
300000 (C119)
C19 C19A C19S C19Y C19*
Normalized Fs

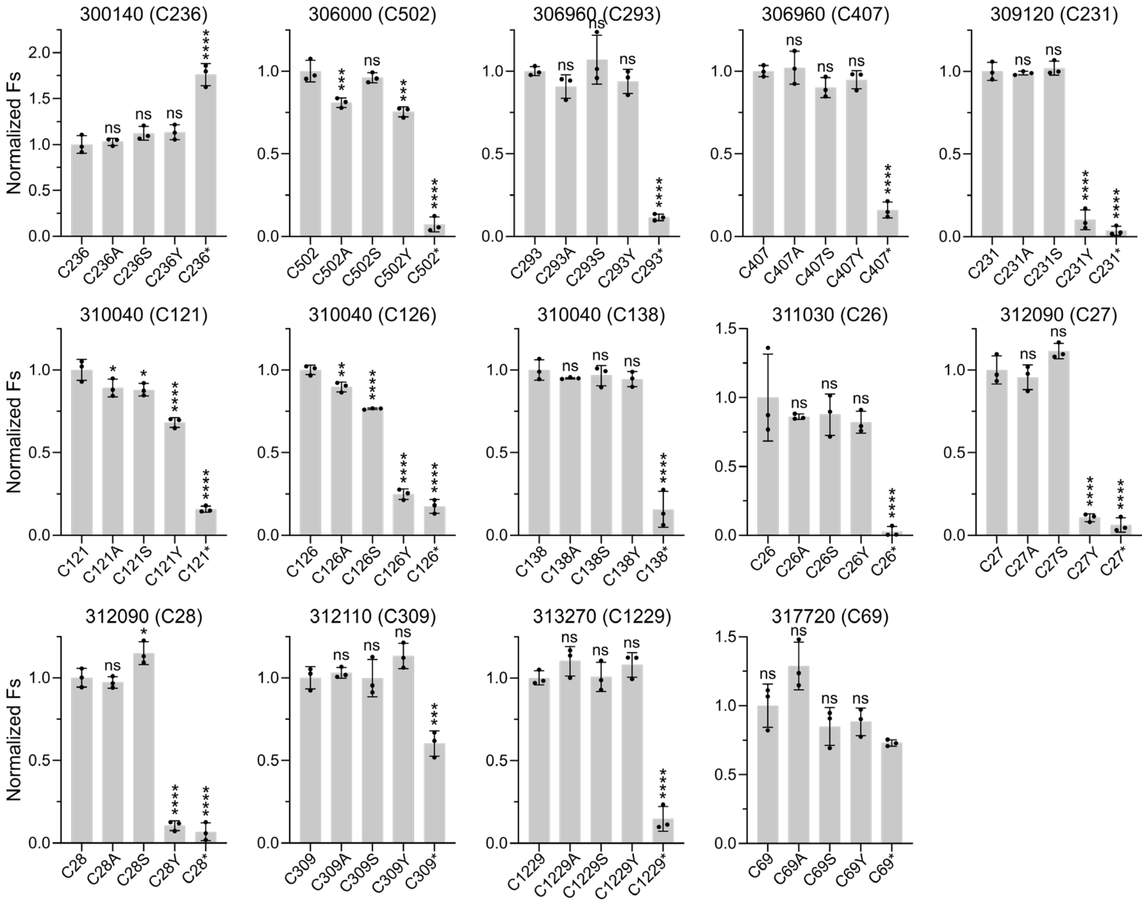

Supplementary Fig. 3: Hyperreactive cysteines in *T. gondii* targeted by CORe exhibit reproducible and diverse mutational profiles. Histograms showing normalized Fs values for five mutations (a recodonized cysteine codon, alanine, serine, tyrosine and stop codon) following mutagenesis of 74 reactive cysteines in *T. gondii* with CORe. The gene identifier (from *Toxo*DB; www.toxodb.org) and associated cysteine residues are annotated above each plot and organised numerically in ascending order. Note that histograms of 227420 (C478) and 257680 (C8/C11) represent those displayed in Figure 2d and are included for completion. Data represents mean ±s.d. for three biological replicates (n=3). Statistical significance of the non-synonymous mutations against the recodonized cysteine control was determined by one-way analysis of variance with Dunnett's corrections for multiple comparisons (*****p* < 0.0001; ****p* < 0.001; ***p* < 0.01; **p* < 0.05, ns = no significance, *p* < 0.05). *p* values: 202820 C20 vs C20A = 0.9998, C20S = 0.0051, C20Y < 0.0001, C20* < 0.0001; 202820 C24 vs C24A = 0.0126, C24S = 0.0001, C24Y < 0.0001, C24* < 0.0001; 202870 C236 vs C236A = 0.0419, C236S = 0.5975, C236Y = 0.2586, C236* = 0.2586; 209950 C65 vs C65A = 0.9999, C65S = 0.9999, C65Y = 0.3040, C65* = 0.3040; 209950 C68 vs C68A = 0.7283, C68S = 0.9877, C68Y < 0.0001, C68* < 0.0001; 215470 C65 vs C65A = 0.0487, C65S < 0.0001, C65Y = 0.0033, C65* = 0.0033; 215470 C73 vs C73A < 0.0001, C73S < 0.0001, C73Y < 0.0001, C73* < 0.0001; 215740 C171 vs C171A = 0.9999,

C171S = 0.3415, C171Y = 0.9766, C171* = 0.9766; 216450 C166 vs C166A = 0.0062, C166S = 0.0004, C166Y < 0.0001, C166* < 0.0001; 216450 C167 vs C167A = 0.0478, C167S = 0.1860, C167Y = 0.0061, C167* = 0.0061; 217900 C90 vs C90A < 0.0001, C90S = 0.7485, C90Y = 0.0034, C90* = 0.0034; 217900 C98 vs C98A = 0.9769, C98S = 0.4439, C98Y = 0.5217, C98* = 0.5217; 219850 C466 vs C466A = 0.8714, C466S = 0.2858, C466Y < 0.0001, C466* < 0.0001; 223620 C81 vs C81A = 0.3570, C81S = 0.9999, C81Y = 0.6413, C81* = 0.6413; 226960 C1222 vs C1222A = 0.9936, C1222S = 0.7889, C1222Y = 0.6484, C1222* = 0.6484; 226970 C60 vs C60A < 0.0001, C60S < 0.0001, C60Y < 0.0001, C60* < 0.0001; 227420 C478 vs C478A < 0.0001, C478S < 0.0001, C478Y < 0.0001, C478* < 0.0001; 228210 C203 vs C203A = 0.8618, C203S = 0.9722, C203Y = 0.8063, C203* = 0.8063; 230450 C155 vs C155A = 0.8692, C155S = 0.9985, C155Y = 0.9998, C155* = 0.9998; 232300 C119 vs C119A = 0.1388, C119S = 0.0126, C119Y = 0.9071, C119* = 0.9071; 233000 C664 vs C664A = 0.3660, C664S = 0.1937, C664Y = 0.4789, C664* = 0.4789; 233140 C154 vs C154A = 0.9021, C154S = 0.6883, C154Y = 0.8542, C154* = 0.8542; 233170 C133 vs C133A = 0.6083, C133S = 0.9999, C133Y = 0.9937, C133* = 0.9937; 236570 C141 vs C141A = 0.9999, C141S = 0.8445, C141Y = 0.9971, C141* = 0.9971; 236570 C200 vs C200A = 0.9999, C200S = 0.9999, C200Y = 0.9961, C200* = 0.9961; 236570 C205 vs C205A = 0.9173, C205S = 0.199, C205Y < 0.0001, C205* < 0.0001; 237820 C80 vs C80A = 0.9088, C80S = 0.9994, C80Y = 0.9661, C80* = 0.9661; 238010 C68 vs C68A = 0.9982, C68S = 0.9984, C68Y = 0.9807, C68* = 0.9807; 238010 C74 vs C74A = 0.3343, C74S = 0.9548, C74Y = 0.4810, C74* = 0.4810; 242330 C54 vs C54A = 0.0799, C54S = 0.5544, C54Y = 0.3031, C54* = 0.3031; 243570 C74 vs C74A < 0.0001, C74S < 0.0001, C74Y < 0.0001, C74* < 0.0001; 243570 C77 vs C77A < 0.0001, C77S < 0.0001, C77Y < 0.0001, C77* < 0.0001; 248320 C285 vs C285A = 0.9437, C285S = 0.9953, C285Y = 0.8754, C285* = 0.8754; 248390 C35 vs C35A = 0.2640, C35S = 0.8727, C35Y = 0.0002, C35* = 0.0002; 249250 C32 vs C32A = 0.9673, C32S = 0.9336, C32Y = 0.4086, C32* = 0.4086; 249900 C276 vs C276A = 0.0631, C276S = 0.003, C276Y < 0.0001, C276* < 0.0001; 251620 C62 vs C62A = 0.8699, C62S = 0.9918, C62Y = 0.9779, C62* = 0.9779; 256970 C257 vs C257A = 0.7769, C257S = 0.0811, C257Y < 0.0001, C257* < 0.0001; 257680 C8 vs C8A = 0.9027, C8S = 0.9998, C8Y = 0.125, C8* = 0.125; 257680 C11 vs C11A = 0.4396, C11S = 0.0236, C11Y = 0.0401, C11* = 0.0401; 258070 C587 vs C587A = 0.1421, C587S = 0.3078, C587Y = 0.5454, C587* = 0.5454; 258970 C2645 vs C2645A = 0.6789, C2645S = 0.9658, C2645Y = 0.9961, C2645* = 0.9961; 260260 C137 vs C137A = 0.7903, C137S = 0.9855, C137Y = 0.9999, C137* = 0.9999; 263300 C257 vs C257A = 0.7371, C257S = 0.7775, C257Y = 0.5327, C257* = 0.5327; 263530 C89 vs C89A = 0.7551, C89S = 0.1788, C89Y = 0.9999, C89* = 0.9999; 266460 C45 vs C45A = 0.0053, C45S = 0.0263, C45Y < 0.0001, C45* < 0.0001; 266990 C945 vs C945A < 0.0001, C945S = 0.5050,

C945Y < 0.0001, C945* < 0.0001; 268835 C240 vs C240A = 0.8650, C240S = 0.8162, C240Y = 0.9806, C240* = 0.9806; 269190 C795 vs C795A = 0.9897, C795S = 0.7903, C795Y = 0.2136, C795* = 0.2136; 269190 C798 vs C798A < 0.0001, C798S < 0.0001, C798Y < 0.0001, C798* < 0.0001; 271888 C6 vs C6A = 0.2276, C6S = 0.2890, C6Y = 0.9603, C6* = 0.9603; 271888 C8 vs C8A = 0.0619, C8S = 0.3543, C8Y = 0.0618, C8* = 0.0618; 276110 C105 vs C105A = 0.3675, C105S = 0.0016, C105Y = 0.0514, C105* = 0.0514; 276110 C106 vs C106A = 0.0007, C106S < 0.0001, C106Y = 0.0252, C106* = 0.0252; 278870 C1746 vs C1746A = 0.9247, C1746S = 0.9990, C1746Y = 0.2461, C1746* = 0.2461; 284560 C63 vs C63A = 0.0781, C63S = 0.9517, C63Y = 0.0059, C63* = 0.0059; 284560 C68 vs C68A = 0.4070, C68S = 0.8718, C68Y = 0.5112, C68* = 0.5112; 297420 C2006 vs C2006A = 0.9990, C2006S = 0.0133, C2006Y = 0.3162, C2006* = 0.3162; 299200 C76 vs C76A = 0.6128, C76S = 0.9990, C76Y = 0.9435, C76* = 0.9435; 300000 C119 vs C119A = 0.7308, C119S = 0.9990, C119Y = 0.4158, C119* = 0.4158; 300140 C236 vs C236A = 0.9838, C236S = 0.3216, C236Y = 0.2492, C236* = 0.2492; 306000 C502 vs C502A = 0.0009, C502S = 0.6202, C502Y = 0.0001, C502* = 0.0001; 306960 C293 vs C293A = 0.4691, C293S = 0.6944, C293Y = 0.7650, C293* = 0.7650; 306960 C407 vs C407A = 0.9819, C407S = 0.2278, C407Y = 0.7168, C407* = 0.7168; 309120 C231 vs C231A = 0.9936, C231S = 0.9484, C231Y < 0.0001, C231* < 0.0001; 310040 C121 vs C121A = 0.0373, C121S = 0.023, C121Y < 0.0001, C121* < 0.0001; 310040 C126 vs C126A = 0.0059, C126S < 0.0001, C126Y < 0.0001, C126* < 0.0001; 310040 C138 vs C138A = 0.7575, C138S = 0.9113, C138Y = 0.6916, C138* = 0.6916; 311030 C26 vs C26A = 0.6812, C26S = 0.7521, C26Y = 0.4927, C26* = 0.4927; 312090 C27 vs C27A = 0.7743, C27S = 0.1165, C27Y < 0.0001, C27* < 0.0001; 312090 C28 vs C28A = 0.899, C28S = 0.0163, C28Y < 0.0001, C28* < 0.0001; 312110 C309 vs C309A = 0.9689, C309S = 0.9999, C309Y = 0.1840, C309* = 0.1840; 313270 C1229 vs C1229A = 0.3435, C1229S = 0.9999, C1229Y = 0.5387, C1229* = 0.5387; 317720 C69 vs C69A = 0.0655, C69S = 0.4534, C69Y = 0.6487, C69* = 0.6487.

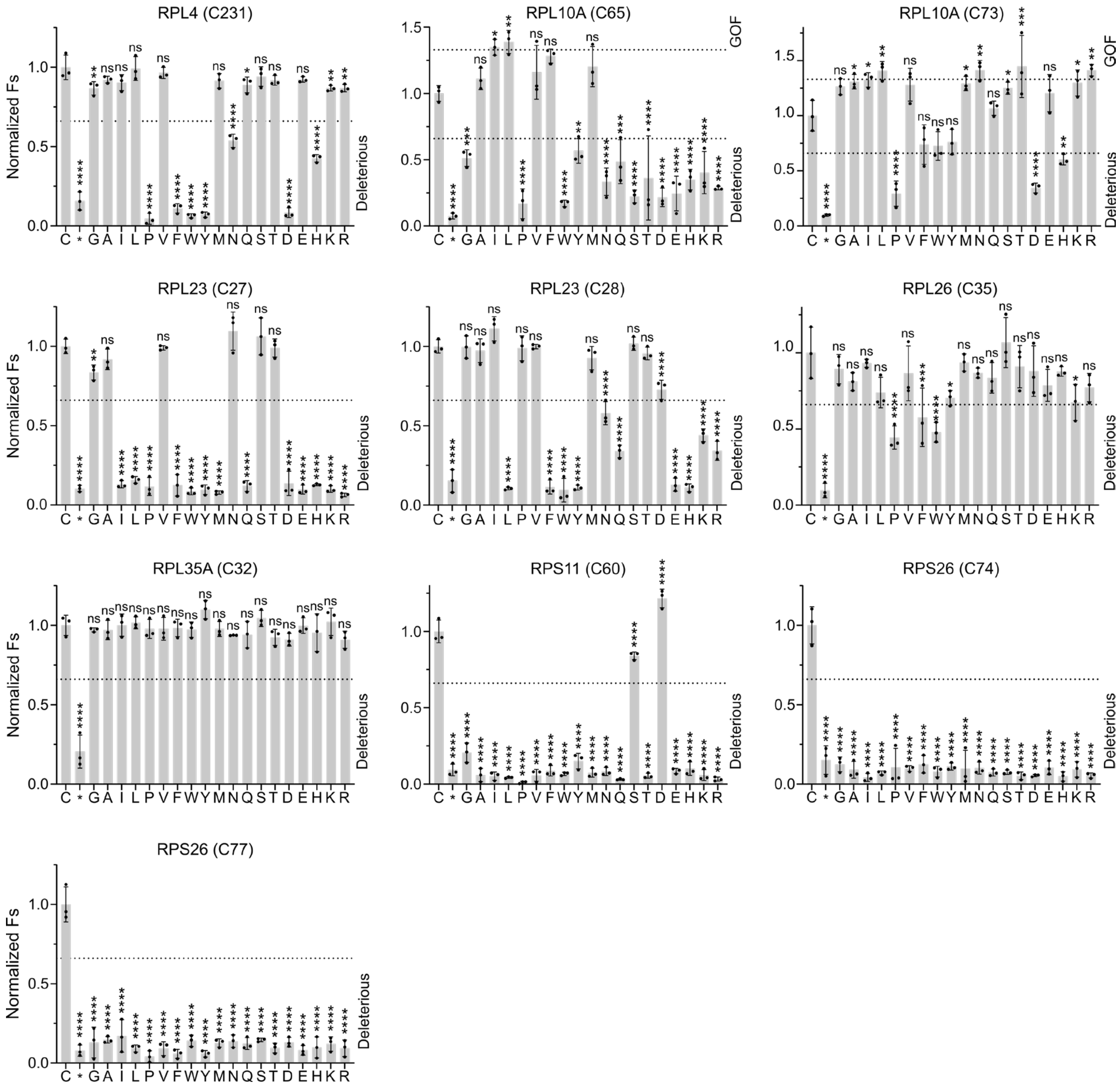

Supplementary Fig. 4: Electrophile-sensitive cysteines of ribosome proteins in *T. gondii* display diverse mutational tolerance. Histograms displaying normalized Fs values for 21 mutations (a recodonized cysteine codon, stop codon and 19 natural amino acid substitutions) following mutagenesis of 10 electrophile-sensitive cysteines in ribosomal proteins with CORe. The gene identifier (from *Toxo*DB; www.toxodb.org) and associated cysteine residues are annotated above each plot and organized numerically in ascending order. Data represents mean ±s.d. for three biological replicates (n=3). Statistical significance of the non-synonymous mutations against the recodonized cysteine control was determined by one-way analysis of variance (****$p < 0.0001$; ***$p < 0.001$; **$p < 0.01$; *$p < 0.05$; ns = non-significant, $p > 0.05$). p values: RPL4 C231 vs C231* < 0.0001, C231G = 0.0059, C231A = 0.261, C231I = 0.0861, C231L = 0.9997, C231P < 0.0001, C231V = 0.9822, C231F < 0.0001, C231W < 0.0001, C231Y < 0.0001, C231M = 0.1758, C231N < 0.0001, C231Q = 0.0316, C231S = 0.6106, C231T = 0.2061, C231D < 0.0001, C231E =

0.2789, C231H < 0.0001, C231K = 0.0063, C231R = 0.0068; RPL10A C65 vs C65* < 0.0001, C65G = 0.0003, C65A = 0.9635, C65I = 0.0151, C65L = 0.0049, C65P < 0.0001, C65V = 0.687, C65F = 0.0797, C65W < 0.0001, C65Y = 0.0014, C65M = 0.3897, C65N < 0.0001, C65Q = 0.0001, C65S < 0.0001, C65T < 0.0001, C65D < 0.0001, C65E < 0.0001, C65H < 0.0001, C65K < 0.0001, C65R < 0.0001; RPL10A C73 vs C73* < 0.0001, C73G = 0.0941, C73A = 0.0307, C73I = 0.0201, C73L = 0.0017, C73P < 0.0001, C73V = 0.0636, C73F = 0.1001, C73W = 0.0722, C73Y = 0.1641, C73M = 0.0486, C73N = 0.0014, C73Q = 0.999, C73S = 0.129, C73T = 0.0005, C73D < 0.0001, C73E = 0.3216, C73H = 0.0023, C73K = 0.042, C73R = 0.0014; RPL23 C27 vs C27* < 0.0001, C27G = 0.007, C27A = 0.481, C27I < 0.0001, C27L < 0.0001, C27P < 0.0001, C27V = 0.9997, C27F < 0.0001, C27W < 0.0001, C27Y < 0.0001, C27M < 0.0001, C27N = 0.2891, C27Q < 0.0001, C27S = 0.809, C27T = 0.9997, C27D < 0.0001, C27E < 0.0001, C27H < 0.0001, C27K < 0.0001, C27R < 0.0001; RPL23 C28 vs C28* < 0.0001, C28G = >0.9999, C28A = 0.9991, C28I = 0.1773, C28L < 0.0001, C28P = 0.9997, C28V = >0.9999, C28F < 0.0001, C28W < 0.0001, C28Y < 0.0001, C28M = 0.6828, C28N < 0.0001, C28Q < 0.0001, C28S = 0.9995, C28T = 0.9873, C28D < 0.0001, C28E < 0.0001, C28H < 0.0001, C28K < 0.0001, C28R < 0.0001; RPL26 C35 vs C35* < 0.0001, C35G = 0.9444, C35A = 0.3606, C35I = 0.9957, C35L = 0.0747, C35P < 0.0001, C35V = 0.7846, C35F = 0.0005, C35W < 0.0001, C35Y = 0.0291, C35M = 0.999, C35N = 0.7975, C35Q = 0.5425, C35S = 0.996, C35T = 0.9861, C35D = 0.8859, C35E = 0.2247, C35H = 0.8678, C35K = 0.0124, C35R = 0.1641; RPL35A C32 vs C32* < 0.0001, C32G = 0.9993, C32A = 0.9992, C32I = >0.9999, C32L = 0.9996, C32P = 0.9994, C32V = 0.9994, C32F = 0.9996, C32W = 0.9993, C32Y = 0.481, C32M = 0.9994, C32N = 0.9358, C32Q = 0.963, C32S = 0.9948, C32T = 0.809, C32D = 0.6001, C32E = >0.9999, C32H = 0.9908, C32K = 0.9994, C32R = 0.594; RPS11 C60 vs C60* < 0.0001, C60G < 0.0001, C60A < 0.0001, C60I < 0.0001, C60L < 0.0001, C60P < 0.0001, C60V < 0.0001, C60F < 0.0001, C60W < 0.0001, C60Y < 0.0001, C60M < 0.0001, C60N < 0.0001, C60Q < 0.0001, C60S < 0.0001, C60T < 0.0001, C60D < 0.0001, C60E < 0.0001, C60H < 0.0001, C60K < 0.0001, C60R < 0.0001; RPS26 C74 vs C74* < 0.0001, C74G < 0.0001, C74A < 0.0001, C74I < 0.0001, C74L < 0.0001, C74P < 0.0001, C74V < 0.0001, C74F < 0.0001, C74W < 0.0001, C74Y < 0.0001, C74M < 0.0001, C74N < 0.0001, C74Q < 0.0001, C74S < 0.0001, C74T < 0.0001, C74D < 0.0001, C74E < 0.0001, C74H < 0.0001, C74K < 0.0001, C74R < 0.0001; RPS26 C77 vs C77* < 0.0001, C77G < 0.0001, C77A < 0.0001, C77I < 0.0001, C77L < 0.0001, C77P < 0.0001, C77V < 0.0001, C77F < 0.0001, C77W < 0.0001, C77Y < 0.0001, C77M < 0.0001, C77N < 0.0001, C77Q < 0.0001, C77S < 0.0001, C77T < 0.0001, C77D < 0.0001, C77E < 0.0001, C77H < 0.0001, C77K < 0.0001, C77R < 0.0001.

Supplementary discussion

Traditional workflows for functional interrogation of amino acids are cumbersome and limited in their multiplexing capacity (**Supplementary Fig. 1**). For a single protein containing a reactive site, the gene must be initially disrupted by gene knockout or knockdown to identify a phenotype-of-interest. This is followed by genetic complementation to assess whether the target gene is necessary and sufficient for the identified phenotype. Following successful wild-type complementation at a genomically distinct locus, reactive site mutants can be similarly tested for their ability to rescue the knockout or knockdown phenotype. Furthermore, if a target gene is known to be essential for cell growth or viability, then any perturbation approach must be conditional. Depending on the target, this process can take many months and culminate in the characterization of sites with not biological function and little therapeutic value. This is further confounded by the requirement to generate and validate multiple complex transfection constructs and cell lines for a single target. This limits the number of residues that can be feasibly handles and interrogated by a researcher. Addressing this challenge, we developed CORe to increase the rate and scale at which amino acids can be functionally annotated. By multiplexing, streamlining, and standardizing various components of the platform, including plasmid assembly, transfections and NGS sample preparation, hundreds of residues can be functionally interrogated in a few weeks (**Supplementary Fig. 1**). However, CORe is limited by its inability to distinguish between protein folding and folding defects upon mutagenesis, or elucidate the specific molecular functions of amino acids, both of which can be assessed using more traditional approaches. While these differences make quantitative comparison of timelines between the two approaches challenging, we anticipate that CORe provides a technological advance that supports existing workflows by allowing researchers to focus detailed molecular characterisations on sites with proven function in cells.

Over recent years, several multiplexed recombineering screens (e.g. MAGE, CREATE) have been developed to simultaneously map the phenotypic effects of thousands of amino acid substitutions across genomes. In these platforms, the allelic frequency of amino acid mutants in a population is monitored over a period of selective pressure or growth, enabling the identification of substitutions that impact cellular fitness. However, these methods estimate mutant abundance by indirectly sequencing episomally-maintained plasmid barcodes, limiting their ability to accurately quantify genotype-phenotype relationships. CORe overcomes these issues by directly sequencing the mutations at the modified chromosomal loci. While several conceptually similar platforms (e.g. CREPE, CasPER) were reported during the development of CORe, their application is restricted to small panels of targets and mutation types. For instance, these technologies use an error-

prone PCR approach to generate libraries of mutational templates, which is limited to single nucleotide changes per codon. This means that typically a maximum of 11 substitution types are readily accessible for a single amino acid. By contrast, CORe uses synthetic oligonucleotide templates that can accommodate any amino acid mutation irrespective of codon distance. In addition, CREPE requires individual CRISPR gRNAs to be pre-screened for high editing efficiency to ensure that mutant growth rates are comparable to wildtype cells. To circumvent the requirement to pre-validate gRNAs, CORe incorporates recodonized sequences into mutational templates to enable enrichment of modified loci by PCR. Here the inclusion of a control template with a wildtype residue at the mutation site enables mutant fitness to be reliably quantified and wild-type normalized even when integration frequencies are low. As demonstrated in preliminary studies with *SAG1*, mutant DNA can be selectively amplified at integration frequencies below 1% (**Extended Data Fig. 9b,c**). Thus, we believe that CORe represents a useful strategy for scalable, direct, quantitative assessment of amino acid function across a diverse range of genomic loci, such as sites identified by RBP.

Previous isoTOP-ABPP studies have implicated cysteine 'hyperreactivity' as a good predictor of functionality in both humans and *C. elegans*. As functional annotation of nucleophilic amino acids is sparse in *T. gondii*, we were unable to reliably assess the relationship between the reactivity and biological function of electrophile-sensitive cysteines identified in this study. However, our data suggest that genes containing electrophile-sensitive cysteines with low and medium reactivity are as likely to be essential for parasite growth as those containing hyperreactive sites (**Fig. 1d**). Together these data suggest that the general characteristic of nucleophilicity or accessibility to electrophilic modification may be the major determinant(s) of cysteine function in this parasite, irrespective of the degree of reactivity. While speculative, this could be related to the enrichment of fitness-conferring cysteines at highly accessible sites on protein surfaces, such as PPI or PTM sites, which possess overall lower reactivity due to greater solvent exposure. For instance, nucleophilic lysines with lower reactivity are known to have greater sensitivity to ubiquitylation and acetylation than hyperreactive sites in protein pockets, presumably due to their increased accessibility to large modifications. Given that a large proportion (~58%) of fitness-conferring cysteines were perturbed exclusively upon tyrosine substitution (**Fig. 3i**), it is conceivable that functional cysteines in the *T. gondii* proteome are overrepresented at PPI interfaces where non-conservative mutations are often required to destabilize high-affinity interactions across multiple residues. Although beyond the scope of this study, further work is clearly required to fully define the functional implications of cysteine reactivity.

Supplementary references

1. Wang, H. H. et al. Programming cells by multiplex genome engineering and accelerated evolution. Nature 460, 894–898 (2009).
2. Garst, A. D. et al. Genome-wide mapping of mutations at single-nucleotide resolution for protein, metabolic and genome engineering. Nature Biotechnology 35, 48–55 (2017).
3. Jakočiūnas, T., Pedersen, L. E., Lis, A. v, Jensen, M. K. & Keasling, J. D. CasPER, a method for directed evolution in genomic contexts using mutagenesis and CRISPR/Cas9. Metabolic Engineering 48, 288–296 (2018).
4. Choudhury, A. et al. CRISPR/Cas9 recombineering-mediated deep mutational scanning of essential genes in Escherichia coli. Molecular Systems Biology 16, e9265 (2020).
5. Weerapana, E. et al. Quantitative reactivity profiling predicts functional cysteines in proteomes. Nature 468, 790–795 (2010).
6. Martell, J. et al. Global Cysteine-Reactivity Profiling during Impaired Insulin/IGF-1 Signaling in C. elegans Identifies Uncharacterized Mediators of Longevity. Cell Chemical Biology 23, 955–966 (2016).
7. Hacker, S. M. et al. Global profiling of lysine reactivity and ligandability in the human proteome. Nature Chemistry 9, 1181–1190 (2017).
8. Engin, H. B., Kreisberg, J. F. & Carter, H. Structure-Based Analysis Reveals Cancer Missense Mutations Target Protein Interaction Interfaces. PLoS One 11, e0152929 (2016).
