# Supplementary material for: CRISPR-based oligo recombineering prioritizes apicomplexan cysteines for drug discovery
Source: Nat Microbiol. 2022 Oct 20;7(11):1891–905. doi: 10.1038/s41564-022-01249-y (PMC9613468; doi:10.1038/s41564-022-01249-y)
Supplement: Supplementary file 2 — Reporting Summary [file 41564_2022_1249_MOESM2_ESM.pdf]

nature portfolio

Corresponding author(s): Matthew A Child, Edward W Tate

Last updated by author(s): Aug 23, 2022

Reporting Summary

Nature Portfolio wishes to improve the reproducibility of the work that we publish. This form provides structure for consistency and transparency in reporting. For further information on Nature Portfolio policies, see our Editorial Policies and the Editorial Policy Checklist.

Statistics

For all statistical analyses, confirm that the following items are present in the figure legend, table legend, main text, or Methods section.

n/a	Confirmed	
[ ]	[x]	The exact sample size (*n*) for each experimental group/condition, given as a discrete number and unit of measurement
[ ]	[x]	A statement on whether measurements were taken from distinct samples or whether the same sample was measured repeatedly
[ ]	[x]	The statistical test(s) used AND whether they are one- or two-sided<br>*Only common tests should be described solely by name; describe more complex techniques in the Methods section.*
[ ]	[x]	A description of all covariates tested
[ ]	[x]	A description of any assumptions or corrections, such as tests of normality and adjustment for multiple comparisons
[ ]	[x]	A full description of the statistical parameters including central tendency (e.g. means) or other basic estimates (e.g. regression coefficient) AND variation (e.g. standard deviation) or associated estimates of uncertainty (e.g. confidence intervals)
[ ]	[x]	For null hypothesis testing, the test statistic (e.g. *F*, *t*, *r*) with confidence intervals, effect sizes, degrees of freedom and *P* value noted<br>*Give P values as exact values whenever suitable.*
[x]	[ ]	For Bayesian analysis, information on the choice of priors and Markov chain Monte Carlo settings
[x]	[ ]	For hierarchical and complex designs, identification of the appropriate level for tests and full reporting of outcomes
[ ]	[x]	Estimates of effect sizes (e.g. Cohen's *d*, Pearson's *r*), indicating how they were calculated

Our web collection on statistics for biologists contains articles on many of the points above.

Software and code

Policy information about availability of computer code

Data collection	NIS-Elements (version 4.60.00) - Visualisation of immunofluorescence micrographs.<br>Image Lab (version 6.1) - Visualisation of western blots and agarose gels.<br>Tecan i-control (version 1.11) - Acquisition of luminescence signal in plate-based in vitro translation assays.<br>AlphaFold (version 2.0) - Generation of tertiary protein structures.<br>Phyre2 (version 2.0) - Generation of tertiary protein structures.<br>FoldX (version 5.0) - Acquisition in silico protein folding stability data.<br>CRISPR-TAPE (version 1.0) - Design of CRISPR sgRNA sequences.
Data analysis	SEQUEST - Searching tandem mass spectrometry data against the Toxoplasma gondii protein database.<br>DTASelect (version 2.0) - Assembling and filtering output peptide sequences from SEQUEST.<br>CIMAGE (version 1.0) - Quantification of isotopic light:heavy ratios from isoTOP-ABPP experiments.<br>BLAST2GO PRO (version 5.0) - Enrichment analysis of functional annotations in the Toxoplasma gondii genome.<br>CASAVA (version 2.17) - Demultiplexing of Illumina indices for next-generation sequencing data analysis.<br>Galaxy (version 21.01.rc1) - Concatenation and splitting of demultiplexed next-generation sequencing data.<br>PyMOL (version 2.1.1) - Alignment and visualisation of tertiary protein structures.<br>ImageJ (version 1.52q) - Processing of immunofluorescence microscopy images.<br>ChemDraw Professional (version 18.0) - Generation of chemical structures.<br>Adobe Illustrator (version 22.1) - Drawing schematics and assembly of final figures.<br>Inkscape (version 0.92.3) - Drawing schematics and assembly of final figures.

GraphPad Prism (version 8.0) - Producing graphs and statistical analyses.
DataWarrior (version 5.5.0) - Filtering chemical compounds by diversity.
Molecular Operating Environment (release 2020.09) - Physicochemical assessment of chemical compounds.

For manuscripts utilizing custom algorithms or software that are central to the research but not yet described in published literature, software must be made available to editors and reviewers. We strongly encourage code deposition in a community repository (e.g. GitHub). See the Nature Portfolio guidelines for submitting code & software for further information.

Data

Policy information about availability of data

All manuscripts must include a data availability statement. This statement should provide the following information, where applicable:

- Accession codes, unique identifiers, or web links for publicly available datasets
- A description of any restrictions on data availability
- For clinical datasets or third party data, please ensure that the statement adheres to our policy

As far as possible all raw data supporting the findings in this study are available within the Article and its Supplementary Information files. Data obtained from sequencing have been deposited in the Sequence Read Archive (SRA; www.ncbi.nlm.nih.gov/sra) under accession number PRJNA860585. The mass spectrometry proteomics data have been deposited to the ProteomeXchange Consortium via the PRIDE partner repository with the dataset identifier PXD035658. Source data are provided with this article. Existing data associated with the gene IDs in this project is available from ToxoDB (https://toxodb.org/toxo/app). Additional unprocessed data is available from the corresponding author upon request.

Human research participants

Policy information about studies involving human research participants and Sex and Gender in Research.

Reporting on sex and gender	N/A
Population characteristics	N/A
Recruitment	N/A
Ethics oversight	N/A

Note that full information on the approval of the study protocol must also be provided in the manuscript.

Field-specific reporting

Please select the one below that is the best fit for your research. If you are not sure, read the appropriate sections before making your selection.

☒ Life sciences ☐ Behavioural & social sciences ☐ Ecological, evolutionary & environmental sciences

For a reference copy of the document with all sections, see nature.com/documents/nr-reporting-summary-flat.pdf

Life sciences study design

All studies must disclose on these points even when the disclosure is negative.

Sample size	For experiments with transgenic mutant parasites, sample sizes were determined based on effect size from previous studies examining mutant phenotypes. For the proteomic profiling of cysteine electrophile-sensitivity sample size was determined following the original application of the isoTOP-ABPP methodology (doi: 10.1038/nature09472). The intention of CORe is to remove bias from sample selection. To that end, CORe was performed for all electrophile-sensitive cysteines that were classified as being 'highly-sensitive' to the iodoacetamide probe with a reactivity ratio <3, and satisfied the criteria of having a published phenotype score <-2 (are fitness-conferring during parasite in vitro growth).
Data exclusions	A single replicate was excluded in the CORe data output for one cysteine mutation (C171Y of TGGT1_215470). The fitness score for this sample (~5.32) was significantly larger than in the two corresponding replicates of the same mutation (~1.17 and ~1.05), and was confirmed by re-sequencing the associated PCR amplicons. Due to the discrepancy of this phenotype between the remaining biological replicates, this data likely reflected a technical issue rather than a genuine physiological response. This data point was therefore considered an outlier and omitted from subsequent statistical analyses.
Replication	Throughout all results in the manuscript were replicated at least twice. Where there was variability between replicates this has been recorded. To ensure biological reproducibility of the phenotypes observed in the CORe mutagenesis screen, transfections and growth assays were performed on distinct days between the three replicates. In addition, the parasites and primary mammalian cells used for different experiments were split and cultured independently. All attempts at replication of the CORe screen were successful.

Randomization	Samples were not randomized but were compared side-by-side with appropriate controls. In CORe experiments, cysteine mutants were generated and assayed on a per-target basis by performing independent transfections and growth assays for each mutagenesis target, avoiding any unanticipated variables that may occur with pooled competitive growth assays using pre-selected target panels.
Blinding	The experiments presented in this study are not subject to experimenter bias and were therefore not blinded.

Reporting for specific materials, systems and methods

We require information from authors about some types of materials, experimental systems and methods used in many studies. Here, indicate whether each material, system or method listed is relevant to your study. If you are not sure if a list item applies to your research, read the appropriate section before selecting a response.

Materials & experimental systems

n/a	Involved in the study
☐	☒ Antibodies
☐	☒ Eukaryotic cell lines
☒	☐ Palaeontology and archaeology
☒	☐ Animals and other organisms
☒	☐ Clinical data
☒	☐ Dual use research of concern

Methods

n/a	Involved in the study
☒	☐ ChIP-seq
☒	☐ Flow cytometry
☒	☐ MRI-based neuroimaging

Antibodies

Antibodies used

Anti-HA High Affinity Rat Monoclonal Antibody (3F10) - Roche, CAT # 11867423001
HA-Tag Rabbit mAb (C29F4) - Cell Signalling Technology, CAT # 3724S, LOT 9
Monoclonal ANTI-FLAG® M2 antibody produced in mouse - Sigma, CAT # F1804
ANTI-FLAG® antibody produced in rabbit - Sigma, CAT # F7425
Toxoplasma gondii SAG1 Monoclonal Antibody (D61S) - Invitrogen, CAT # MA5-18268, LOT TB2525582
Toxoplasma gondii Rabbit Polyclonal Antibody (PA1-7252) - Invitrogen, CAT # AB_561769
Alexa Fluor™ 488 Goat anti-Mouse IgG (H+L) - Invitrogen, CAT # A11001
Alexa Fluor™ 594 Goat anti-Mouse IgG (H+L) - Invitrogen, CAT # A11032, LOT 1985399
Alexa Fluor™ 488 Goat anti-Rabbit IgG (H+L) - Invitrogen, CAT # A11034, LOT 1885241
Alexa Fluor™ 594 Goat anti-Rabbit IgG (H+L) - Invitrogen, CAT # A11012
Goat anti-Mouse IgG (H+L) Secondary Antibody, HRP (62-6520) - Invitrogen, CAT # 31430
Goat anti-Rabbit IgG (H+L) Secondary Antibody, HRP - Invitrogen, CAT # 31460

Validation

Anti-HA High Affinity Rat Monoclonal Antibody (3F10) is a commercial antibody used for immunodetection of proteins tagged with the hemagglutinin epitope YPYDVPDYA. In their application note, the manufacturers validated this antibody for labeling against a HA-tagged GST protein. Using western blot, we have demonstrated labeling of a HA-tagged hypothetical protein expressed in T. gondii parasites, which is not observed in parental (untransfected) cell lines.

HA-Tag Rabbit mAb (C29F4) is a commercial antibody for immunodetection of exogenously expressed proteins containing the hemagglutinin epitope (YPYDVPDYA). The manufacturers have validated this antibody against a range of HA-tagged proteins across a broad range of applications including western blot, immunohistochemistry and flow cytometry. Using immunofluorescence microscopy, we have demonstrated labeling of HA-tagged hypothetical protein expressed in T. gondii parasites, which is not observed in parental (untransfected) cell lines.

Monoclonal anti-FLAG M2 produced in mouse (F1804) is a commercial antibody used for immunodetection of proteins containing the FLAG epitope (DYKDDDDK). Using immunofluorescence, we have demonstrated labelling of FLAG-tagged RPL4 constructs in T. gondii, which is not seen in the parental cell line. This antibody has been previously validated for western blots, immunofluorescence and flow cytometry applications in many peer-review articles and organisms, including T. gondii (e.g. doi 10.1038/srep03199 and 10.1371/journal.pbio.1001358).

ANTI-FLAG® antibody produced in rabbit (F7425) is a polyclonal antibody used for immunodetection of proteins tagged with the FLAG epitope (DYKDDDDK). The manufacturers have validated this antibody against a range of FLAG-tagged proteins and applications including immunoprecipitation of FLAG-BAP fusion protein from E. coli crude lysate, indirect immunofluorescence in HEK293T cells expressing FLAG-JNK, and western blot in E. coli periplasmic extract. This antibody has also previously been validated in T. gondii, with peer-reviewed publications reporting its use (e.g. doi 10.1371/journal.ppat.1001232).

Toxoplasma gondii SAG1 Monoclonal Antibody (D61S) is a commercially available antibody routinely used as a surface marker stain for T. gondii tachyzoites and has been validated in several peer-review articles (e.g. doi 10.1128/mBio.01678-17 and 10.3389/fcimb.2017.00259).

Toxoplasma gondii Polyclonal Antibody (PA1-7252) is produced in rabbit for specific immunodetection of RH stain Toxoplasma gondii parasites. It is routinely raised and used as a control antibody for parasite surface marker istaining n immunofluorescence applications or loading controls in western blot (e.g. doi 10.7554/eLife.36491).

Eukaryotic cell lines

Policy information about cell lines and Sex and Gender in Research

Cell line source(s)	HFF-1 ATCC® SCRC-1041™, HEK293F (Thermo Scientific CAT R79007), Toxoplasma gondii and Plasmodium falciparum strains (various)
Authentication	ATCC certified (HFF), Thermo Scientific certified (HEK293F), in-house tested (Toxoplasma and Plasmodium)
Mycoplasma contamination	Confirmed free via monthly PCR testing in-house
Commonly misidentified lines (See ICLAC register)	No commonly misidentified lines were used.
