# Supplementary figures and images for: CRISPR-based oligo recombineering prioritizes apicomplexan cysteines for drug discovery
Source: Nat Microbiol. 2022 Oct 20;7(11):1891–905. doi: 10.1038/s41564-022-01249-y (PMC9613468; doi:10.1038/s41564-022-01249-y)

*Tg*ISPH (C478)

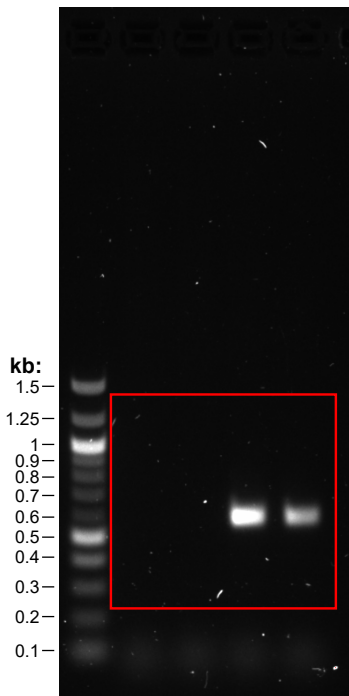

*Tg*MLC1 (C8)

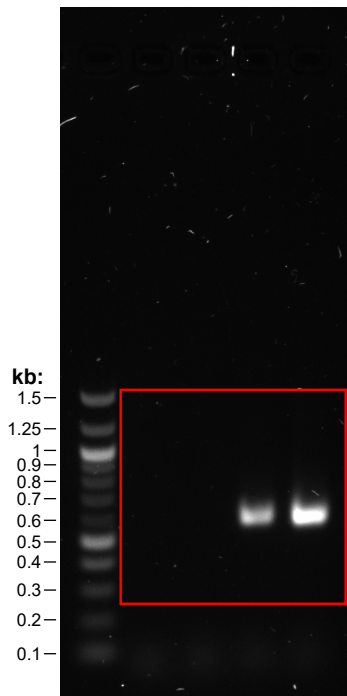

*Tg*MLC1 (C11)

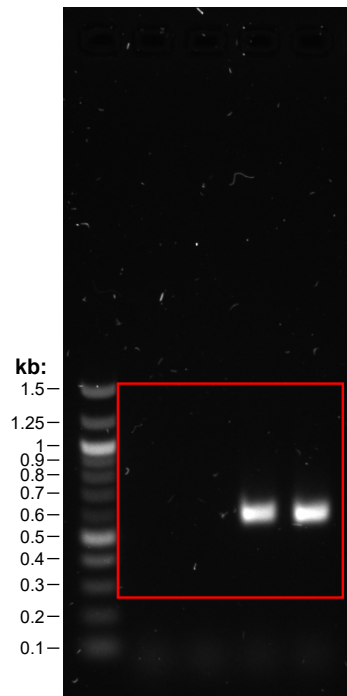

Supplement: Source Data Fig. 2 — Unprocessed agarose gels. [file 41564_2022_1249_MOESM6_ESM.pdf]

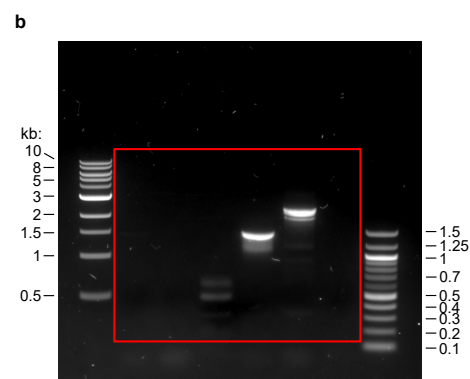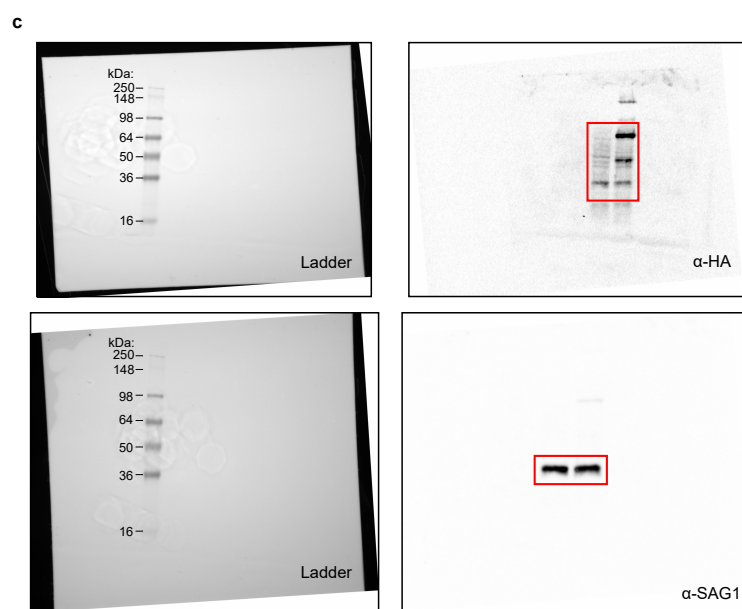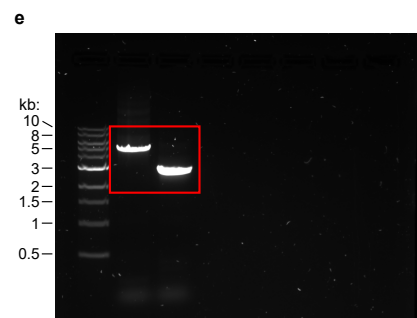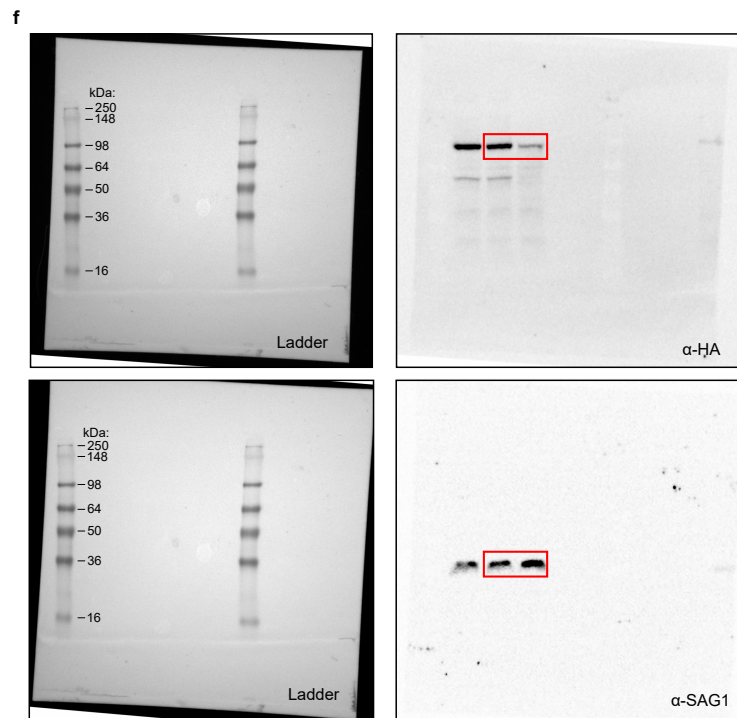

Supplement: Source Data Extended Data Fig. 2 — Unprocessed agarose gels and western blots. [file 41564_2022_1249_MOESM11_ESM.pdf]

**c**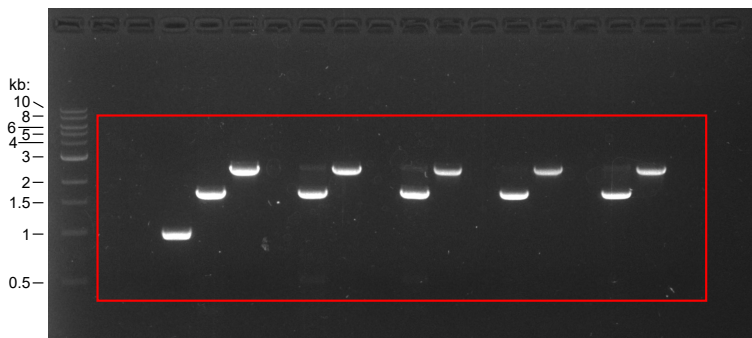**f**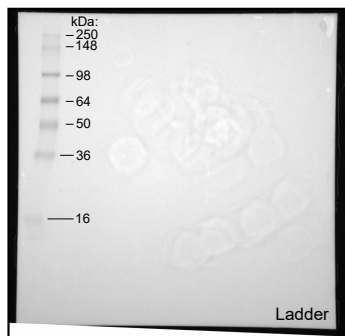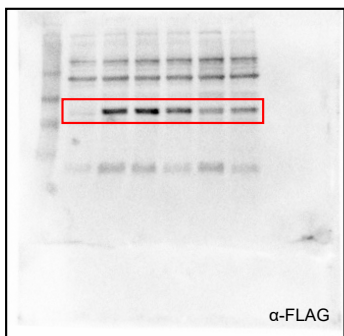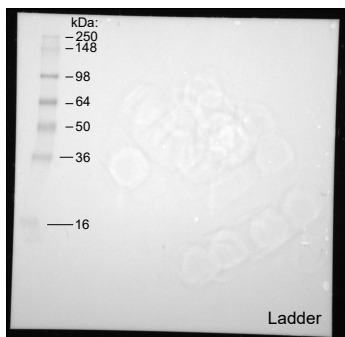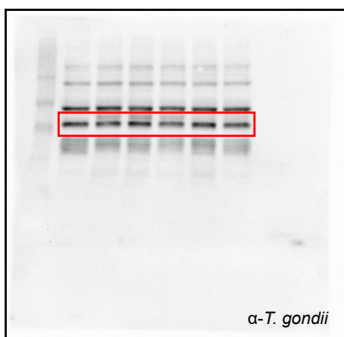

Supplement: Source Data Extended Data Fig. 8 — Unprocessed agarose gels and western blots. [file 41564_2022_1249_MOESM16_ESM.pdf]
